# An improved power loss model of full-bridge converter under light load condition

**DOI:** 10.1371/journal.pone.0208239

**Published:** 2018-12-06

**Authors:** Lele Yao, Donghui Li, Lingling Liu

**Affiliations:** School of Electrical and Information Engineering, Tianjin University, Tianjin, China; University of Science and Technology Beijing, CHINA

## Abstract

When the full-bridge converter works under the light load condition, the power efficiency obtained by the theoretical model is much different from that of the actual converter. Facing with this situation, an improved power loss model based on the typical power loss model is proposed. In this paper, the typical power loss model is called typical model for short and the improved power loss model is called proposed model for short. Firstly, the antiparallel freewheeling diodes at the arms of full-bridge circuit are taken into account. Every barrier junction capacitance of Schottky diode in the rectifier circuit is neglected. Then, the turning-off loss of full-bridge and the core loss of inductive components (the transformer and the filter inductor) in the typical model are compensated and modified by combining the theoretical values with the measured input current under the minimum and the maximum output current. In addition, it also corrects the equivalent resistance related to the conduction loss of converter. Eventually, the proposed model is established. The rise time and fall time of the midpoint voltage of two arms, and the fluctuation degree of the reverse bias voltage related to the Schottky rectifier diodes are regarded as the local indexes. The conduction time of the metal oxide semiconductor field effect transistor (MOSFET) in each switching period, and the power efficiency of converter are regarded as the global indexes. Based on the analyses, the local indexes are compared qualitatively, while the global indexes are compared quantitatively. It is found that the differences of the local indexes between the proposed model and the experiment are smaller than those between the typical model and the experiment. Meanwhile, the global indexes of the proposed model are closer to the experimental results. Therefore, it can be further demonstrated that the proposed model is more approximate to the actual converter than the typical model.

## 1 Introduction

DC-DC converters have been paid much attention to in recent years. They can be widely applied in many fields such as photovoltaic (PV) system, electric vehicle (EV) system, and so on. In the PV system, the maximum power point tracking (MPPT) methods are developed by the DC-DC converter. Regulating duty cycles of main switches is an effective way to realize the MPPT. Besides the conventional perturbation and observation method and the incremental conductance method, some advanced MPPT algorithms have been successfully used. For example, an improved MPPT method combined with characteristics of solar cell arrays under the shadow condition has been proposed in [1]. In [2], a method without algorithm-specific parameters has been improved and it is also called natural cubic-spline-guided Jaya algorithm (S-Jaya). In [3], the nonsimplified single-diode model is used to determine the MPP. Curve fitting method is fully developed in the proposed real-time estimation of the MPP. In [4], the emulation of PV module is obtained according to the mathematical model of PV cell. Several characteristic curves of certain PV system and related MPPT methods can be achieved at the output terminals of proposed system. In the EV system, different energy storages are the source or load of DC-DC converter and power is converted by the converter. In [5], an interleaved-boost full-bridge DC-DC three-port converter is designed. The phase angle and duty cycle are variables in the modulation. The duty cycle is related to the power flow between two inputs and the phase angle is relevant to the output voltage. In [6], a three-phase rectifier, a three-phase inverter, and a dual-active bridge DC-DC converter are taken as a whole. Dynamic efficiency models of the whole are established so that the proposed varied dc-link voltage method can be effectively developed. In [7], a new bidirectional DC-DC converter is proposed and it is connected to the dual battery energy source and the DC-bus of different voltage levels. Based on the hybrid model of converter, different modes of power flow can be regulated. Similarly, the DC-DC converter has been popular in the electric aircraft system. In [8], a fuel cell, a battery, and a supercapacitor are connected to the DC bus by the proposed quadruple active bridge converter. Furthermore, the energy sources and the power flow can be individually controlled by using multiple DC-DC converters. In the DC grid system, the DC-DC converter is also the main part of power transfer device. In [9], the modular multilevel converter is the investigated object and the switch-based model of insulated gate bipolar transistor (IGBT) is established. Transient performance of switches is reflected by the dynamic model which is based on the curve-fitting. Therefore, the power loss of converter can be analyzed and calculated. In [10], wind energy and solar energy are the two sources in the grid system. The voltage and frequency are regulated according to the droop characteristics of system. The practical conditions have been fully considered. Furthermore, both of the two energy blocks can achieve the MPPT.

From what has been introduced above, it can be concluded that different DC-DC converters play important roles in many fields and their models can help researchers have knowledge of converters.

As the typical representative, full-bridge DC-DC converters with isolated transformer have been widely applied in the new energy generation, server power supply, and electric vehicles [11–13]. The work environment of the converters is becoming complex. As a kind of power conversion and transmission device, power efficiency is an important measurement index. Especially, under no more than 10% load, the efficiency has been paid much attention to. In order to study the improvement methods of power efficiency under the light load condition, many scholars rely on the approximate equivalent power loss models of full-bridge converters [14]. It is not difficult to find that there is a logical difference between the theoretical analyses and the actual results according to the appropriate power loss model, which can be taken as an effective and reasonable reference to improve power efficiency under light load. On the contrary, when the power loss model is not accurate, it will cause much large difference between the theoretical analyses and the actual results. Furthermore, it is not conducive to obtaining the methods of improving efficiency under light load.

The power loss is mainly resulted from the switching loss and the conduction loss when the full-bridge converter works under light load condition. Therefore, equivalent power loss model contains the two kinds of losses. Switching loss model mainly involves the IGBT or MOSFET at the arm of full-bridge, and the Schottky diode or MOSFET in the rectifier circuit. In [15], the nonlinear junction capacitances between the gate, drain, and source are taken into account in the equivalent model of MOSFET. Meanwhile, the parasitic inductance is also the consideration. Descriptions of state equation are given because the characteristics of MOSFET in the process of turning-on and turning-off are combined. In [16], the influence of load current on miller platform of MOSFET is considered in the equivalent model. Furthermore, the dead time of two bridges can be effectively adjusted. In [17], the thermodynamic model of switches at two bridges is established for the phase-shift full-bridge converter. The model is related to switching loss. In [18], the equivalent power loss model is obtained by converting the primary side and secondary side of transformer. The model of switches is approximately coped so that the range of zero-voltage switching (ZVS) for MOSFETs can be effectively analyzed. In [19], the voltage and current of IGBT are sampled and reconstructed in a specific way. Average switching loss model is obtained when the values of sample and reconstruction are simply calculated. In [20–21], the full-bridge converter contains the synchronous rectifier. Each junction capacitance between drain and source of MOSFET is taken as a linear capacitor in the model. Dead time of lagging bridge is regulated by load current. The difference between [20] and [21] is the current mode of filter inductor. The current works continuous mode so that filter inductor can be treated as a current source in [20]. Contrarily, the current works discontinuous mode and only the filter capacitor is regard as a voltage source in [21].

Conduction loss model mainly involves the core loss and copper loss of the inductive components. On the other hand, the conduction loss of switches can not be neglected. In [22], the leakage inductance, the excitation inductance, and the inter layer distributed capacitance are estimated according to the actual structure of isolated transformer in the power loss model. It can be used to analyze the core loss and copper loss of full-bridge converter. In [23], it is considered that the voltage generated at the midpoints between two arms of bridge is square wave rather than sine wave. Therefore, the modified Steinmetz equation is adopted when the core loss of transformer in the model is analyzed. In [24], the BH curve of isolated transformer is divided under the asymmetrical square wave excitation. Estimated core loss can be much close to the measured value based on the corrected core loss model. In [25], the BH curve of isolated transformer is fully used when the optimal phase shift angle of full-bridge converter is considered under light load. Furthermore, core loss model is optimized on the bases of revised Steinmetz equation. In [26], the space structure between the transformer and the synchronous rectifier is focused on so that the core loss and copper loss can be further clarified. Values of the leakage inductance and excitation inductance can be effectively obtained by combining with the operating points of LLC resonant circuit. In [27], the passive components including isolated transformer are idealized and the ideal model of converter can be established. The overall conduction loss model is qualitatively analyzed on the bases of simulation and experiment. In [28], the influence of conduction resistance related to the switches is taken into account in the equivalent model of full-bridge converter. The DC resistance of inductive components and the equivalent series resistance (ESR) of filter capacitor are also in the proposed model. In [29], the converter is equivalent to the RL circuit which represents the overall losses. The power loss model includes the switching loss, the conduction loss of IGBTs, and the loss resulted from forward voltage drop of rectifier diodes. In [30], both sides of isolated transformer are converted. The AC resistance of winding at both sides of transformer and the equivalent AC resistance of filter circuit represent the overall conduction losses of full-bridge converter in the power loss model.

Reference [31] has certain representativeness. Every junction capacitance between drain and source of MOSFET at both sides of transformer is replaced by the linear capacitance in the switching loss model. The values of junction capacitance and the switching time are both taken from the datasheet of relevant MOSFET. In [31], the conduction loss contains the loss come from resistance of switches, and the core loss and copper loss of inductive components. The core loss is calculated according to the typical Steinmetz equation and the copper loss is calculated according to the DC resistances in the conduction loss model. Though the power loss model is reasonable and it has been applied in the aforementioned literatures, there are still some problems in analyzing the power efficiency of actual converter.

1) The parameters of switches at the arms of full-bridge and the parameters of diodes in the rectifier circuit all come from the corresponding datasheets. These parameters are measured under certain conditions, rather than be suitable for any experimental conditions.

2) The fast recovery diodes treated as reverse freewheeling are antiparallel with the related switches at the actual arms of full-bridge circuit. However, existing literatures do not consider the effect of the diodes.

3) The core loss of the inductive components is derived from traditional Steinmetz equation which is suitable for sinusoidal excitation. The core loss is also obtained according to the modified Steinmetz equation, which involves change rate of magnetic induction intensity in real time. The later way is relatively complicated.

4) The DC resistance of the winding is directly selected according to the equivalent resistance of inductor and isolated transformer. Skin effect of winding under high frequency is not taken into account.

In order to simplify the calculation of manual operation, the power loss model of full-bridge converter under light load is fully analyzed by using the Saber software. Two power loss models are established in this paper. The first model corresponds to the typical model, and the second model corresponds to the proposed model. The typical model is derived from the [31], and the proposed model is obtained by improving the typical model. The reference has proved that it is feasible and effective to adopt the pulse width modulation (PWM) for full-bridge converter under light load condition. So the PWM strategy is applied in the two models and the actual converter. Investigation is developed according to the fact that the input voltage, the output voltage, the output current, and the switching frequency in the two models are all the same that in the actual converter. The proposed model is established by considering the antiparallel freewheeling diodes at the arms of full-bridge and ignoring the barrier junction capacitances of rectifier diodes. Meanwhile, it needs to be compensated and corrected the turning-off loss of MOSFETs, the equivalent core loss resistance of inductive components, and the equivalent conduction loss resistance of converter. The whole aforementioned modified parts come from the typical model. Furthermore, the local indexes and global indexes are formulated. The theoretical results of the two models are compared with the experimental results according to the indexes, so that the approximation degree between the two power loss models and the actual converter can be measured. There are four sections in this text. The principle of the two models is formulated in the first section. Aided analyses and design based on the Saber software are shown in the second section. The actual full-bridge converter is used to verify the theoretical results in the third section. Furthermore, the comprehensive analyses on the results and the comparisons of indexes are presented in the fourth section. Whether the proposed model is closer to the actual converter than the typical model is judged by a series of analyses and verifications.

## 2 Formulation of power loss model

**Fig 1:**
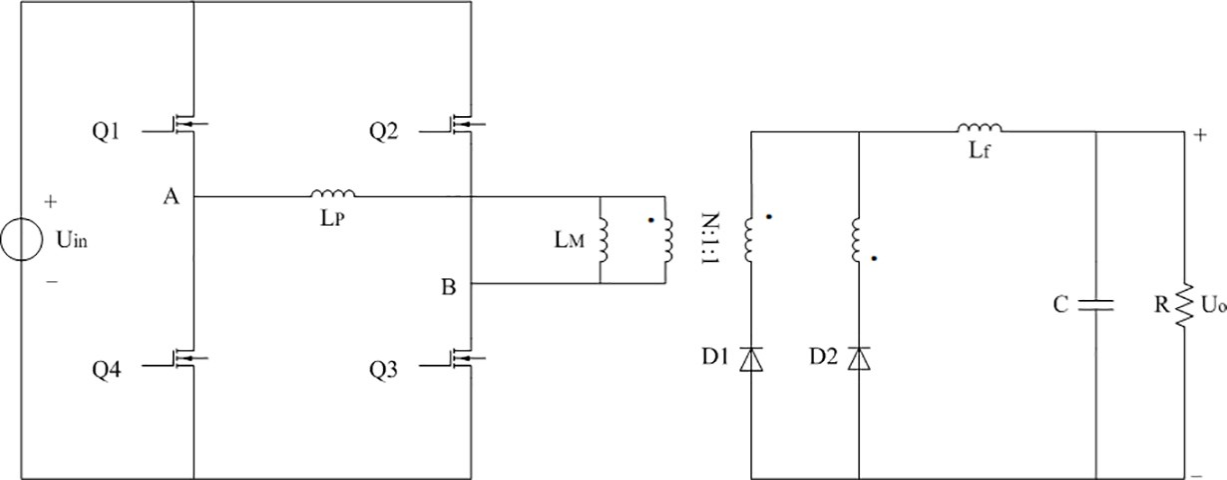
Schematic diagram of full-bridge converter.

The full-bridge converter studied in this paper is shown in Fig 1. The constant voltage source U_in_ represents the input voltage. Q1 to Q4 stand for the certain type of MOSFET. L_P_ and L_M_ represent the leakage inductance and excitation inductance of isolated transformer respectively. The turns ratio between primary side and two secondary sides is N:1:1 in the transformer. D1 and D2 mean the specific type of Schottky rectifier diodes. L_f_, C, and R indicate the filter inductor, filter capacitor, and load respectively. The output voltage of converter is always regulated to U_o_.

The reference [31] has confirmed that the PWM strategy is feasible and effective for the full-bridge converter under light load condition. So the PWM strategy is adopted in this paper. In addition, continuous current mode (CCM) of the filter inductor L_f_ is regarded as the research background which is corresponding to the 1% load to 10% load.

### 2.1 Typical model

**Fig 2:**
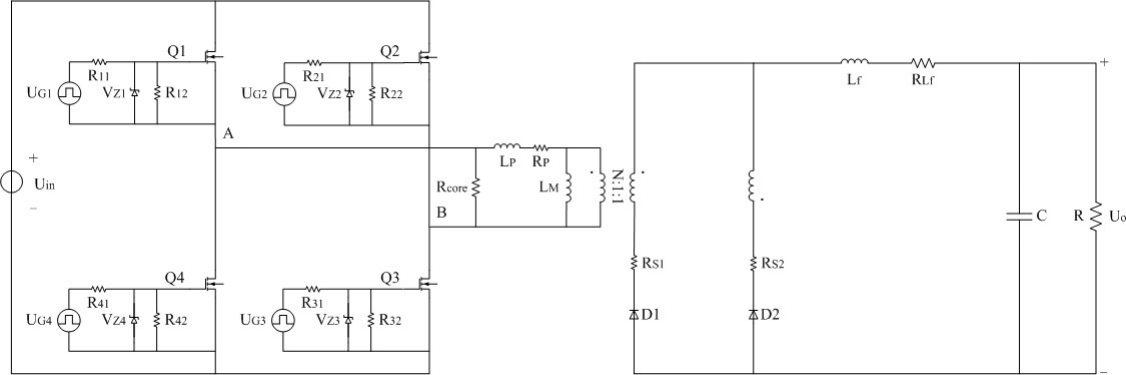
Typical model. (A) Estimation of R_P_, R_S1_, R_S2_, and R_core_.

In order to establish the power loss model of Fig 1 under light load, the typical model shown in Fig 2 can be obtained on the bases of [31]. Same parts of Fig 1 will be removed in the next description. U_Gi_ represents the driving signal of corresponding MOSFET and the subscript “i” is counted from one to four. R_i1_ and R_i2_ are the driven resistors of related MOSFET and the subscript “i” is counted from one to four. V_Zi_ means the certain type of zener diode of relevant MOSFET and the subscript “i” is counted from one to four. R_core_ indicates the equivalent core loss resistance of isolated transformer and filter inductor. R_P_, R_S1_, and R_S2_ are the equivalent copper loss resistances of winding at two sides of isolated transformer. R_Lf_ is the equivalent copper loss resistance of the filter inductor L_f_.

#### (A) Estimation of R_P_, R_S1_, R_S2_, and R_core_

The approximate estimates of equivalent copper loss resistances R_P_, R_S1_, R_S2_, and the estimates of equivalent core loss resistance R_core_ are presented as follows.

**R**_**P**_**:** Assume that the resistance coefficient with winding in the transformer is *ρ*. The length of corresponding winding is *L*. The corresponding winding is wrapped by enameled wire whose strands are *x*. The radius of enameled wire is *r*. Assume that the resistance referred to every share of enameled wire is R_P1_. R_P_ is equivalent to the parallel resistors whose the number is *x*. Each parallel resistor is the R_P1_ and it is listed as follows:

$$R_{P1}=\frac{\rho L}{\pi r^{2}}.$$ (1)

The solving process of R_S1_ and R_S2_ is similar to that of R_P_.

**R**_**core**_**:** One of the inductive components is taken an example. Assume that the initial time of each switching period *T* is *t*_0_, and the switching frequency is treated as *f*. Instantaneous voltage is *u*(*t*). The initial value of magnetic induction intensity is *B*_0_ and the peak value is Δ*B*, which the instantaneous value is expressed as *B*(*t*). The effective cross sectional areas and effective volumes of skeleton are *S* and *V* respectively. The number of turns is *n*. The empirical constants corresponding to the magnetic core are *κ*, *α*, and *β*. The *α* is usually taken from one to two and the *β* is usually taken from two to three. The average value of core loss per unit volume is expressed as *p*_core_. Therefore, the average expression of integral core loss *P*_core(i)_ is calculated as follows:

$$B(t)=B_{0}-\frac{1}{nS}\int_{t_{0}}^{t}u(\tau )d\tau$$

$$\Delta B=\underset{t_{0}\to t_{0}+T}{\max}[B(t)]-\underset{t_{0}\to t_{0}+T}{\min}[B(t)]$$

$$p_{core}=\kappa f^{\alpha }\Delta B^{\beta }$$

$$P_{core(i)}=p_{core}V$$

$$P_{core}=\sum P_{core(i)}.$$

**Fig 3:**
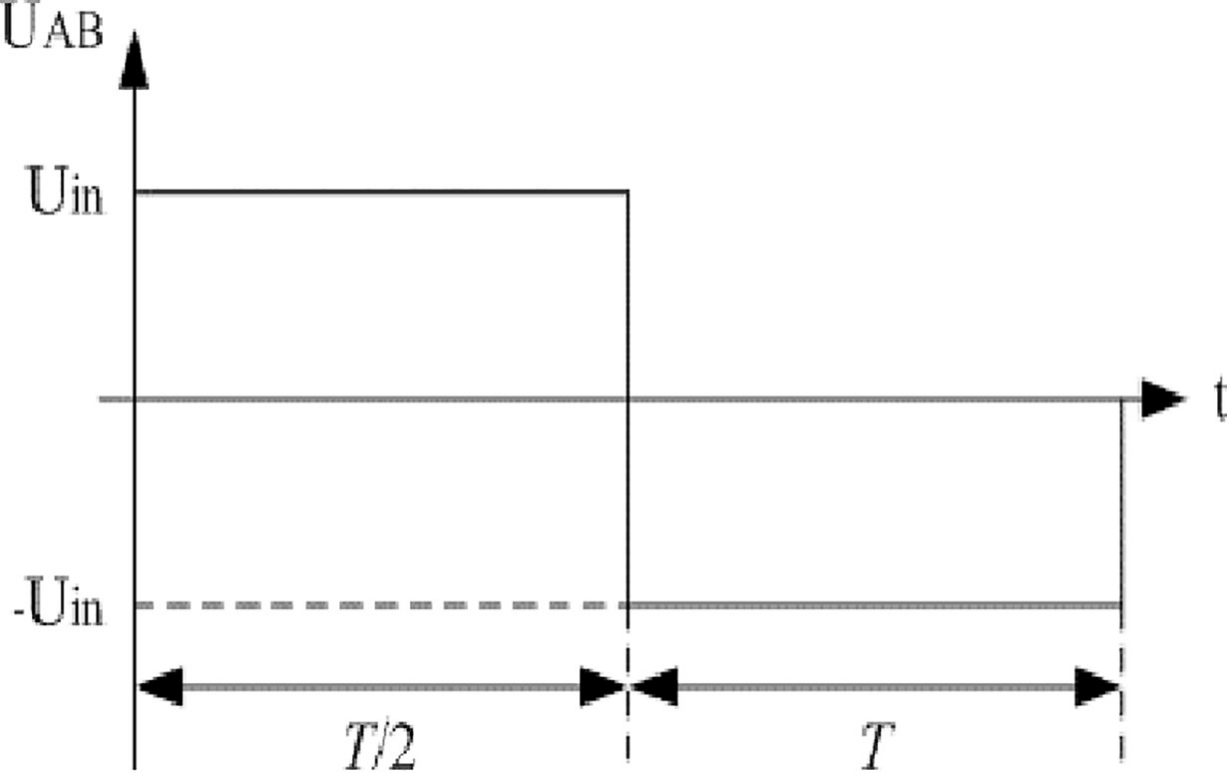
Estimation of U_AB_.

**Fig 4:**
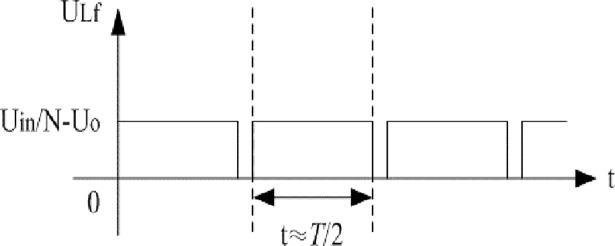
Estimation of U_Lf_.

Based on the voltage characteristics of inductive components, voltage U_AB_ can be approximately represented by Fig 3. Similarly, voltage U_Lf_ can be approximately illustrated as Fig 4.

In summary, R_core_ can be approximated according to the following expression:

$$R_{core}=\frac{U_{in}^{2}}{P_{core}}.$$ (2)

#### (B) Characteristics of typical model

The typical model mainly has the following characteristics.

1) The parameters of MOSFETs and Schottky diodes are directly applied the default values according to the corresponding actual datasheets.

2) The core loss of inductive components is estimated by the traditional Steinmetz equations, and the corresponding equivalent core loss resistance can be obtained.

3) The copper loss of inductive components is directly calculated according to the DC resistance of relevant winding.

### 2.2 Proposed model

#### (A) Proposed modeling method

For the sake of making the proposed model more accurate to reflect the actual power efficiency than the typical model, it is necessary to correct the core loss of inductive components and the switching loss of full-bridge circuit on the bases of typical model. It is known that switches at the arms of full-bridge can realize zero voltage turning-on when the inductor at primary side of transformer is large. The switching loss is almost dominated by the turning-off loss because of the large leakage inductance. Furthermore, turning-off loss of these switches is studied in this paper.

Assume that the range of output current for the converter is from *I*_o(min)_ to *I*_o(max)_. The theoretical input power is *P*_in1_ and the theoretical output power is *P*_o1_ in the typical model. The experimental input power is *P*_ine_ and the experimental output power is *P*_oe_. Q1 is taken as an example. The average turning-off voltage, the average turning-off current, and the turning-off duty cycle are shown as *U*_off_, *I*_off_, and *D*_off_ respectively under the theoretical analyses on the typical model and proposed model. The turning-off loss of Q2, Q3, and Q4 are all the same that of Q1.

When the output current is *I*_o(min)_, the following expressions are presented:

$$\Delta P_{core}=\frac{\left(P_{ine(1)}-P_{oe(1)})-(P_{in1(1)}-P_{o1(1)}\right)}{2}$$ (3)

$$4U_{off(1)}I_{off(1)}D_{off(1)}k_{1}=\frac{\left(P_{ine(1)}-P_{oe(1)})-(P_{in1(1)}-P_{o1(1)}\right)}{2}$$

where the *U*_off(1)_, *I*_off(1)_, and *D*_off(1)_ are the theoretical *U*_off_, *I*_off_, and *D*_off_ in the typical model when the output current is *I*_o(min)_. *P*_ine(1)_, *P*_oe(1)_, *P*_in1(1)_, and *P*_o1(1)_ are the *P*_ine_, *P*_oe_, *P*_in1_, and *P*_o1_ under the *I*_o(min)_.

When the output current is *I*_o(max)_, the following expression is shown:

$$4U_{off(2)}I_{off(2)}D_{off(2)}k_{2}=[(P_{ine(2)}-P_{oe(2)})-(P_{in1(2)}-P_{o1(2)})]-\Delta P_{core}$$

where the *U*_off(2)_, *I*_off(2)_, and *D*_off(2)_ are the theoretical *U*_off_, *I*_off_, and *D*_off_ in the typical model when the output current is *I*_o(max)_. *P*_ine(2)_, *P*_oe(2)_, *P*_in1(2)_, and *P*_o1(2)_ are the *P*_ine_, *P*_oe_, *P*_in1_, and *P*_o1_ under the *I*_o(max)_.

The coefficients *k*_1_ and *k*_2_ satisfy the following equations:

$$0.1a+b=k_{1}$$ (4)

$$a+b=k_{2}.$$ (5)

Eq (3) represents compensation of core loss in the improved method. The *a* and *b* in (4) and (5) are the compensating factors of turning-off loss in the proposed model of full-bridge converter.

In the proposed model, the modified equivalent core loss resistance R_core_ and the modified equivalent turning-off loss resistance R_switch_ are expressed respectively as follows:

$$R_{core}=\frac{U_{in}^{2}}{P_{core}+\Delta P_{core}}$$ (6)

$$P_{switch(I_{o})}=4(aI_{o}+b)U_{off(I_{o})}I_{off(I_{o})}D_{off(I_{o})}$$ (7)

$$R_{switch(I_{o})}=\frac{U_{in}^{2}}{P_{switch(I_{o})}}.$$ (8)

*P*_core_ in (6) is namely the core loss shown in (2). *I*_o_ in (7) is the output current of converter. *R*_switch(Io)_ in (8) is namely the R_switch_ under the *I*_o_. *U*_off(Io)_, *I*_off(Io)_, and *D*_off(Io)_ are the theoretical *U*_off_, *I*_off_, and *D*_off_ in the proposed model when the output current is *I*_o_.

**Fig 5:**
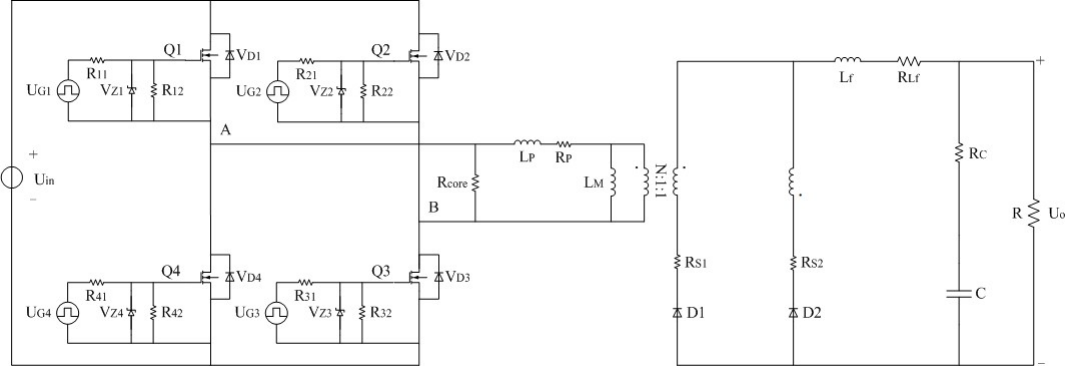
Proposed model without R_switch_.

**Fig 6:**
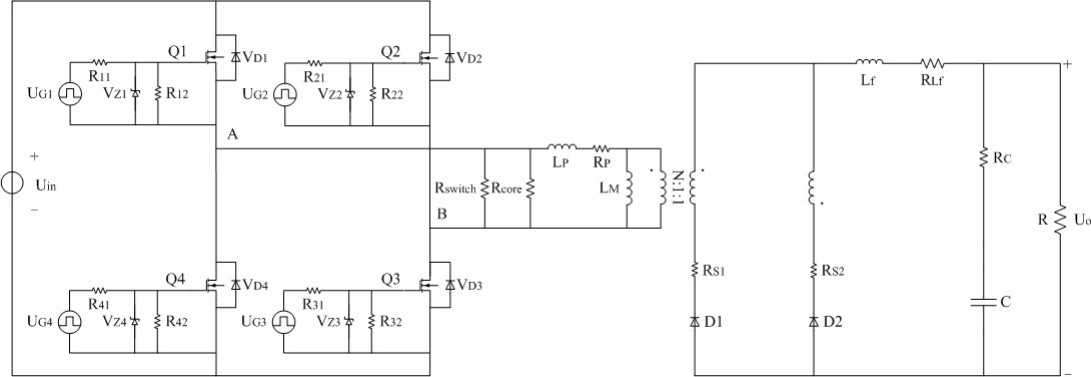
Proposed model.

The proposed model is divided into two steps due to the need to estimate equivalent resistance R_switch_. The first step is shown in Fig 5 and the second step is presented in Fig 6. There are five differences between the Fig 5 and the Fig 2. For the Fig 5, the differences are listed as follows.

1) The certain type of freewheeling diodes V_D1_ to V_D4_ are antiparallel with their corresponding MOSFETs Q1 to Q4.

2) The barrier junction capacitances of the rectifier diodes D1 to D2 are neglected.

3) The equivalent core loss resistance R_core_ is obtained by the (6).

4) The equivalent copper loss resistances R_P_, R_S1_, and R_S2_ are calculated according to the skin effect of winding. The equation can be seen in (9).

5) The influence resulted from the ESR of filter capacitor C is considered.

Turning-off loss of switches at the arms of full-bridge is compensated on the bases of Fig 5. The corresponding equivalent resistance R_switch_ is obtained according to the (7) and (8). Furthermore, the complete proposed model is shown in Fig 6. The Fig 5 and Fig 6 are the same except for the R_switch_. Power efficiency of proposed model is calculated according to the Fig 6.

#### (B) Estimation of R_P_, R_S1_, and R_S2_

The approximate estimates of equivalent copper loss resistances R_P_, R_S1_, and R_S2_ are listed as follows.

**R**_**P**_**:** Assume that the resistance coefficient with winding in the transformer is *ρ* and the unit is Ω.m/mm^2^. The length of corresponding winding is *L* and the unit is m. The corresponding winding is wrapped by enameled wire whose strands are *x*. The radius of enameled wire is *r* and the unit is mm. The frequency of current in the corresponding winding is *f* and the unit is Hz. Assume that resistance referred to each strand of enameled wire is R_P1_. R_P_ is equivalent to the parallel resistors whose the number is *x*. Each parallel resistor is the R_P1_ and it is listed as follows:

$$R_{P1}=\frac{\rho L}{\pi [r^{2}-(r-\frac{66.1}{\sqrt{f}}{)}^{2}]}.$$ (9)

The solving process of R_S1_ and R_S2_ is similar to that of R_P_.

#### (C) Characteristics of proposed model

Similarly, the proposed model has the following characteristics which are different from the typical model.

1) The corresponding antiparallel freewheeling diode of MOSFET is considered. Switching loss is characterized by the related equivalent resistance in the main circuit.

2) The influence resulted from the barrier junction capacitance of Schottky rectifier diode is neglected.

3) Eq (3) is directly applied to estimate and compensate the core loss of inductive components on the foundation of typical model and experiment. Furthermore, the (6) is applied to obtain the equivalent core loss resistance.

4) According to the skin effect, equivalent copper loss resistance of inductive components is represented by the AC resistance of winding.

5) The ESR of the filter capacitor is applied in the proposed model.

### 2.3 Applicability of proposed model

In this paper, other devices such as IGBT can be applied in the proposed model. It can be explained according to the compositions of equivalent circuit and the proposed modeling method.

The aforementioned subsection “Proposed model” is mainly the proposed modeling method. It is essentially a comprehensive method by combining boundary measurement with linear compensation. The following formulation is the description of the compositions of equivalent circuit.

In Fig 5 and Fig 6, proposed model is illustrated by the equivalent circuit of converter. It mainly contains driving circuit and main circuit. For the full-bridge converter, the main switches are MOSFET and IGBT. Meanwhile, the rectifier devices are usually Schottky diodes and sometimes they are MOSFETs. The rest parts of equivalent circuit are transformer, filter inductor, and filter capacitor respectively. The main switches and related driving circuits are taken as a whole unity. They are used to generate the square wave voltage in the main circuit and it is the source of switching loss. On the other hand, no matter what rectifier devices are, they are just the diodes with low conduction loss essentially. Transformer, filter inductor, and filter capacitor are the source of core loss and conduction loss. The power conversion is determined by the transformer and the majority of aforementioned losses come from the transformer.

Based on the Fig 5, the Fig 6, and the light load condition, the switching loss and the core loss are the two important considerations. Therefore, the main switches and corresponding driving circuits, and the transformer are the key compositions of equivalent circuit respectively. They are listed as follows in brief.

(1) Main switches and corresponding driving circuits

**Fig 7:**
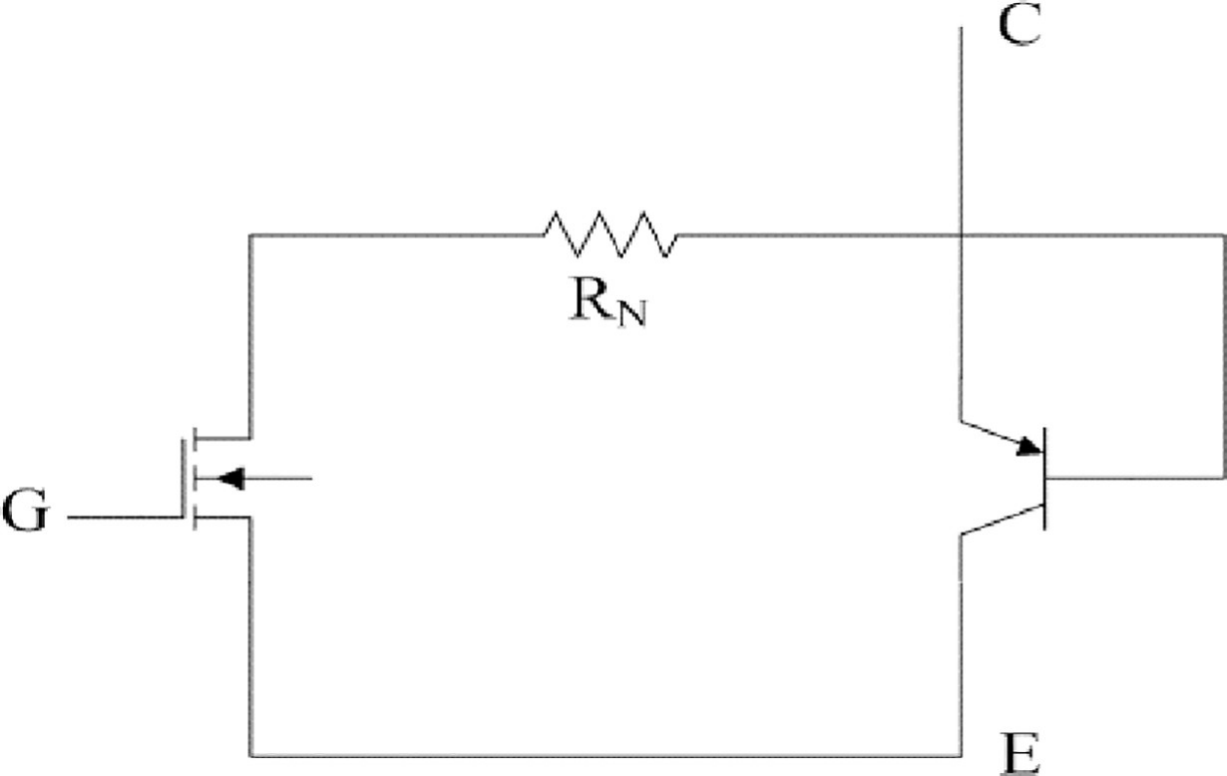
Equivalent circuit of IGBT.

**Fig 8:**
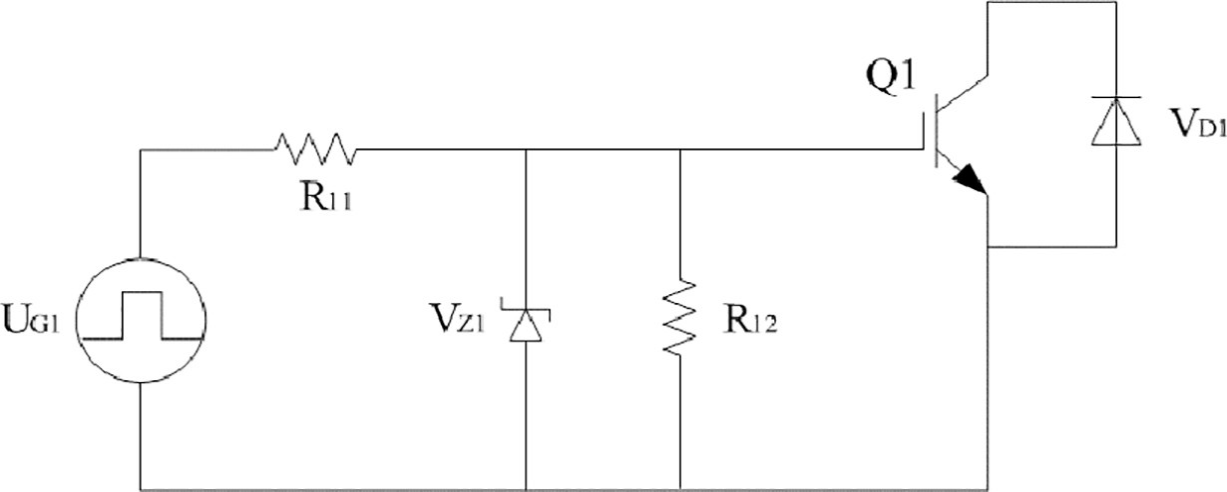
Related driving circuit.

When the main switch is MOSFET, every driving circuit is shown in Fig 5 and Fig 6. They are almost same except the timing of pulse sources Q1 to Q4. Similarly, when the main switch is IGBT, the equivalent circuit of IGBT and related driving circuit can be seen in Fig 7 and Fig 8.

No matter what the main switch is, parameters of MOSFET or IGBT are directly applied with the default values according to the actual datasheet. R_switch_ in Fig 6 represents the switching loss of main switches and it plays an important role in the equivalent circuit. R_switch_ is obtained according to the comprehensive modeling method by combining boundary measurement with linear compensation. Considered the definition of switching loss, the proposed method does not need much prior knowledge and it is not limited to specific main switches.

(2) Transformer

**Fig 9:**
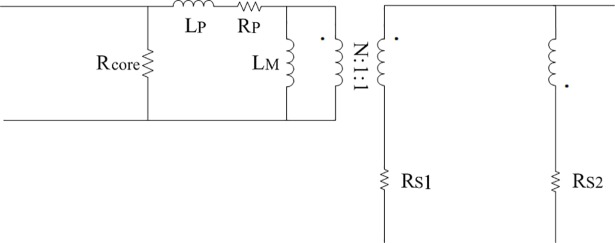
Equivalent circuit of transformer.

The equivalent circuit of transformer can be seen in Fig 9. It mainly includes the leakage inductance, the magnetizing inductance, the equivalent core loss resistance and the equivalent copper loss resistances.

In this paper, the leakage inductance and magnetizing inductance are obtained by the short-circuit test and open-circuit test. The equivalent copper loss resistances are calculated according to the AC resistances of winding. The equivalent core loss resistance comes from the inductive components and the main source of core loss is transformer. Under the light load condition, the R_core_ in Fig 5 and Fig 6 also plays an important role in this equivalent circuit and it is modified by the boundary measurement which does not need much prior knowledge.

In summary, R_switch_ and R_core_ are the important compositions of equivalent circuit. The solving process of them is the combination of boundary measurement and linear compensation. Furthermore, the proposed model which is based on the comprehensive method does not need much prior knowledge. Therefore, the model is not limited to the application of specific components.

## 3 Aided analyses by the Saber software

### (A) Main parameters

The following formulations are all derived from the practical converter. As shown in Fig 1, the type of switches Q1 to Q4 is IRFP460. The type of Schottky rectifier diodes is V50100PW. The leakage inductance L_P_ is 600μH and the excitation inductance L_M_ is 11.5mH. The turns ratio between the primary side and the two secondary sides is 8.42:1:1. The filter inductor L_f_ is 250μH and the filter capacitor C is 470μF. U_Gi_ in Fig 2 and Fig 6 is a square wave excitation that the amplitude is 15V and the frequency is 24kHz. R_i1_ and R_i2_ are 6.2Ω and 10kΩ respectively in the two models. The type of V_Zi_ in the two models is IN4744A. The type of V_Di_ in the proposed model is MUR3060WT. All the aforementioned subscript “i” is counted from one to four. When the average output voltage U_o_ is equal to 12V, the approximation degree between the two models and the actual converter is verified by investigating the following cases respectively.

Case 1: U_in_=115V and I_o_=0.1A.

Case 2: U_in_=120V and I_o_=0.2A, 0.3A, …, and 1A.

The current under full load is 10A.

In order to reduce the amount of manual calculation, the Saber software is fully used to analyze the two models. The Q1 to Q4 and V_Z1_ to V_Z4_ in the two models, and V_D1_ to V_D4_ in the proposed model are directly called from the corresponding types in the principle library of Saber. Furthermore, the whole parameters are default according to the actual datasheets. The D1 and D2 are applied with their respective SPICE models as follows.

### (B) SPICE model of rectifier diode

Assume that the forward instantaneous voltage and current of each rectifier diode are *v* and *i* respectively. The reverse voltage of each rectifier diode is *v*_r_. The barrier junction capacitance of each rectifier diode is *C*_j_. The following parameters are involved in the SPICE model constructed by (10) and (11). These parameters include the reverse saturation current *I*_S_, the emission coefficient *N*, the voltage equivalent of temperature *V*_T_, the zero bias capacitance *C*_j0_, the junction voltage *V*_j_, and the capacitance gradient factor *M*:

$$i=I_{S}(e^{\frac{v}{NV_{T}}}-1)$$ (10)

$$C_{j}=\frac{C_{j0}}{{\left(1-\frac{v_{r}}{V_{j}}\right)}^{M}}.$$ (11)

According to the datasheet of V50100PW, the (10), and the (11), aforementioned parameters in the SPICE model can be solved.

### (C) Formulation of the local indexes and global indexes

**Fig 10:**
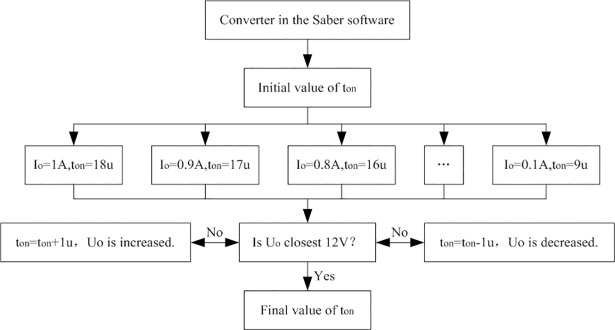
Determination process of t_on_.

PWM strategy is adopted in the full-bridge converter and the switching frequency is 24kHz. Conduction time of each MOSFET in each switching period is defined as t_on_ whose unit is μs. The t_on_ is namely the duration time that high level of U_Gi_ in one period under both aided analyses and experiment. The subscript “i” is also counted from one to four. For convenience of analysis, t_on_ is chosen as an integer. The determination process of t_on_ is shown in Fig 10.

**Fig 11:**
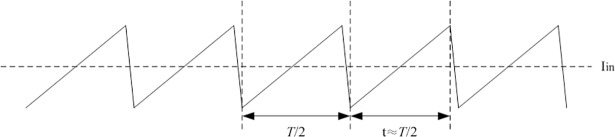
Diagram of input current.

When the input voltage, output voltage, and output current of full-bridge converter are given, the power efficiency is obtained according to the input current. In this paper, the input current of converter can be illustrated as Fig 11 under both aided analyses and experiment. As can be seen from Fig 11, the I_in_ represents the average input current. For each period of input current, the rise time of instantaneous current is approximately close to the half of switching period *T*/2. Assume that the output voltage is U_o_ and the load resistor is R. The power efficiency *η* can be formulated by (12):

$$\eta =\frac{U_{o}^{2}}{U_{in}I_{in}R}.$$ (12)

The local indexes and the global indexes are given as follows so that the approximation degree between the two models (typical model and proposed model) and the actual converter can be measured respectively. In terms of the proposed model, the local indexes are designated according to the judgment standard for the rationality of adding the antiparallel freewheeling diodes at the arms of full-bridge and neglecting the barrier junction capacitance of each Schottky diode in the rectifier circuit. In terms of the two models, the global indexes are designated according to the rationality that the equivalent turning-off loss resistances, the influence of equivalent core loss resistance of inductive components, and the influence of equivalent conduction loss resistances are all considered.

#### Local indexes

When the levels of output current I_o_ are 0.1A, 0.5A, and 1A respectively, the related characteristics of key waveforms corresponding to the typical model, the proposed model, and the experiment are listed as follows.

1) Rise time and fall time of U_AB_. (The time is defined as the transition time between the positive amplitude and the negative amplitude in this paper.)

2) Fluctuation degree of the reverse bias voltage related to the D1 and D2.

#### Global indexes

When the levels of output current I_o_ are 0.1A, 0.2A, …, and 1A respectively, the global indexes related to the typical model, the proposed model, and the experiment are listed as follows.

1) t_on_: Conduction time of each MOSFET in each switching period.

2) η: Power efficiency of converter.

### 3.1 Typical model

#### (A) Calculated parameters

In Fig 2, the known default parameters are excluded and other parameters are as follows.

1) Eq (1) is solved according to the actual parameters of winding. These parameters can be seen in the appendix. We can know that R_P_, R_S1_, R_S2_, and R_Lf_ are 0.2496Ω, 0.0127Ω, 0.0127Ω, and 0.0333Ω respectively.

2) The fitting coefficients of ferrite for inductive elements given by manufacturer are as follows. Other relevant parameters can be seen in the appendix. Eq (2) is combined with Fig 3 and Fig 4. We can know that R_core_ is 17.42kΩ when U_in_ is 115V and R_core_ is 17.044kΩ when U_in_ is 120V. The aforementioned fitting coefficients are presented:

κ=2.2429,α=1.55,andβ=2.5214.

3) The undetermined parameters of SPICE model related to the D1 and D2 are listed as follows:

$$Is=451.69nA,\;N=1,\;V_{T}=26mV,\;C_{j0}=3nF,\;V_{j}=1V,\;and\;M=0.64.$$

#### (B) Presentation of theoretical results

According to the Fig 2 and the aforementioned parameters, the typical model is established in the Saber software. When the levels of output current I_o_ are 0.1A, 0.5A, and 1A respectively, the following waveforms can be seen.

**I_o_ = 0.1A**

**Fig 12:**
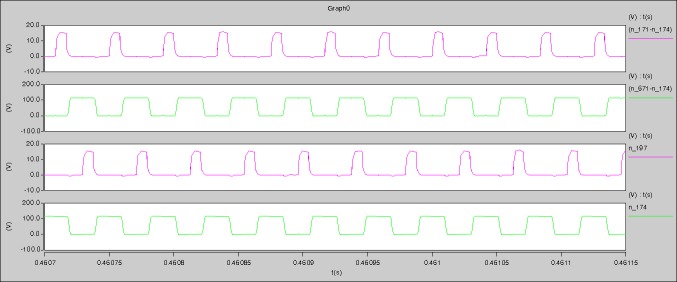
Waveforms under theoretical analysis.

**Fig 13:**
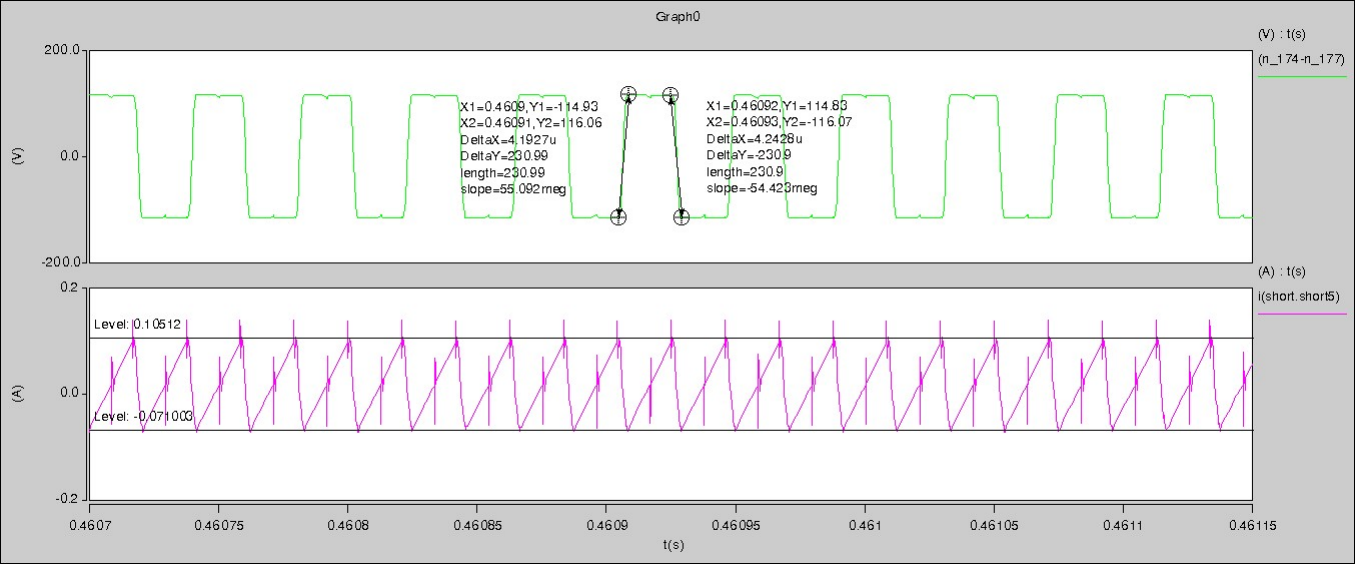
Waveforms under theoretical analysis.

**Fig 14:**
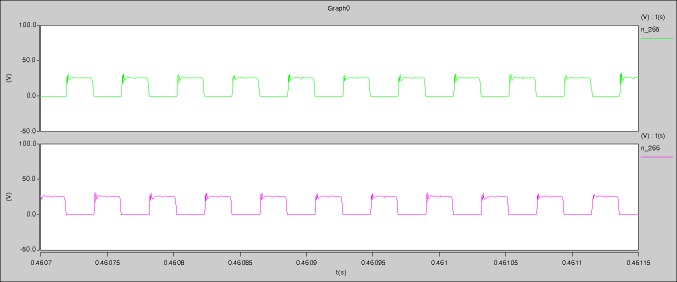
Waveforms under theoretical analysis.

**Fig 15:**
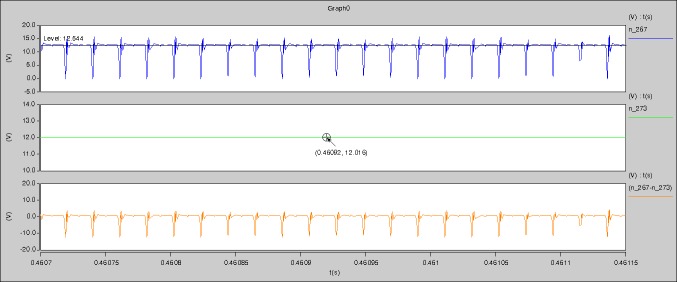
Waveforms under theoretical analysis.

**Fig 16:**
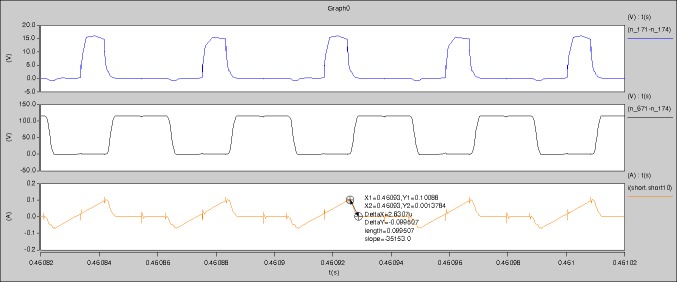
Waveforms under theoretical analysis.

The waveforms under theoretical analyses can be seen from Figs 12–16.

The meanings of Fig 12 from top to bottom are the gate-source voltage of Q1, the drain-source voltage of Q1, the gate-source voltage of Q4, and the drain-source voltage of Q4 respectively.

The meanings of Fig 13 from top to bottom are the midpoint voltage of two arms, and the input current respectively.

The meanings of Fig 14 from top to bottom are the reverse voltage of D1, and the reverse voltage of D2 respectively.

The meanings of Fig 15 from top to bottom are the total voltage corresponding to the secondary side of transformer and the Schottky rectifier diode, the output voltage, and the voltage of filter inductor respectively.

The meanings of Fig 16 from top to bottom are the gate-source voltage of Q1, the drain-source voltage of Q1, and the branch current of Q1 respectively.

Based on Figs 12–16, the following results can be obtained.

1) The drain-source voltage of Q1 and Q4 can be estimated as zero when they are turning-on.

2) The approximate amplitude of the midpoint voltage of two arms is 115.0V. And the approximate amplitude of the voltage of filter inductor is 1.0V.

3) The input current can be treated as triangular waveform and the rise time is close to the half of switching period *T*/2.

4) The average input current and the output voltage are 0.0171A and 12.016V.

5) The rise time and fall time of U_AB_ are 4.1927μs and 4.2428μs.

6) The fluctuation degree of the reverse bias voltage related to the D1 and D2 is large.

7) The conduction time of each MOSFET in each switching period t_on_ is 8μs.

**Fig 17:**
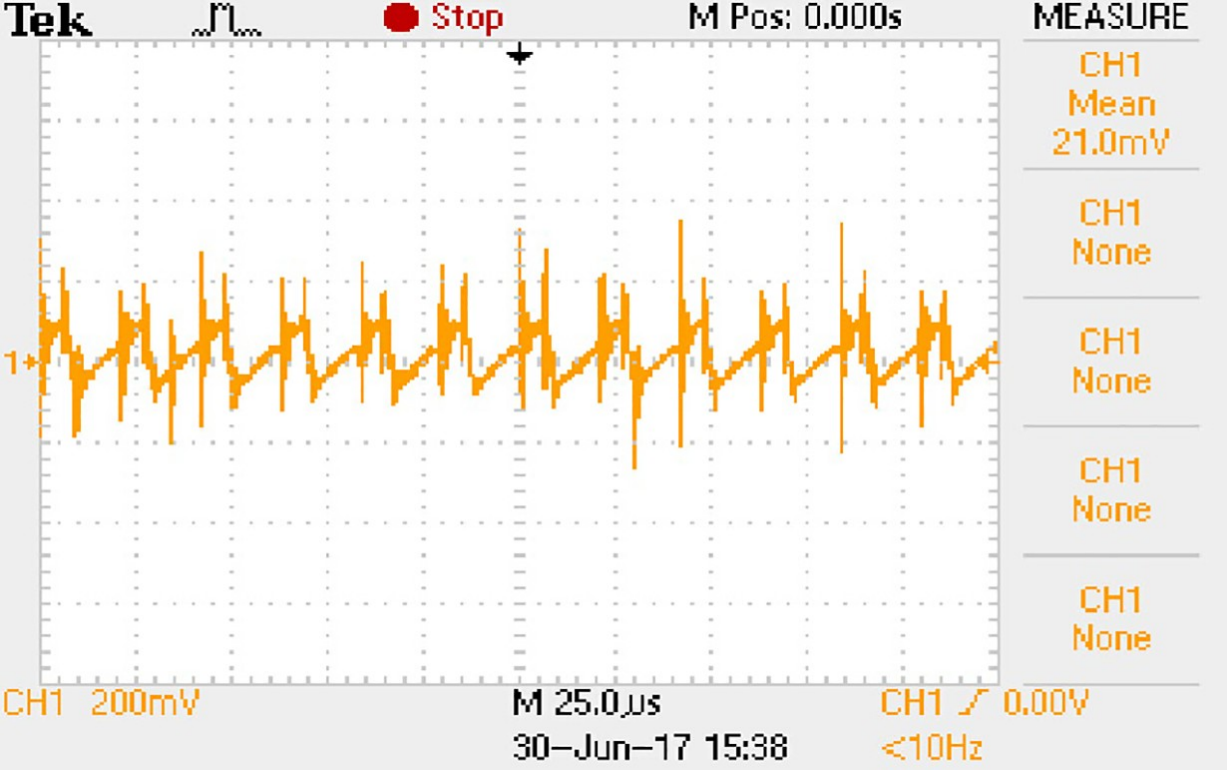
Actual measured input current.

In order to solve (6) and (8) in the proposed model, it is necessary to obtain the actual average input current when the level of output current I_o_ is 0.1A. The experimental waveform of input current can be seen in Fig 17.

Fig 17 is the voltage waveform of 1Ω sampling resistor, so it can reflect the input current directly. The measured method with Tektronix oscilloscope TDS2012B is formulated as follows.

1) The sensitivity of time axis is always 25μs/div, and the sensitivity of longitudinal axis is gradually adjusted from 50mV/div to the large range until the following requirement is reached. The largest sensitivity of longitudinal axis is 5V/div.

2) At each sensitivity of longitudinal axis, 30 sets of waveforms are measured. Then the average values and mean square deviations are calculated. When the mean square deviation is within 3I_o_ which the unit is mV, the measurement objective is reached. For example, the mean square deviation cannot exceed 3mV when the I_o_ is 1A.

3) At the final sensitivity of longitudinal axis, the waveform whose average value is most approximate to the calculated average value is chosen as the typical input current of converter.

Based on Fig 17, the following results can be obtained.

1) The input current can be treated as triangular waveform and the rise time is close to the half of switching period *T*/2.

2) The average input current is 0.0210A.

**I_o_ = 0.5A**

**Fig 18:**
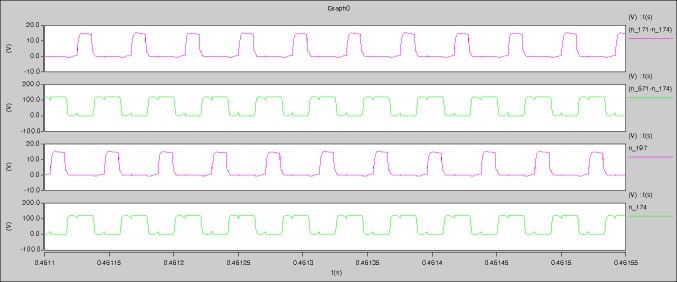
Waveforms under theoretical analysis.

**Fig 19:**
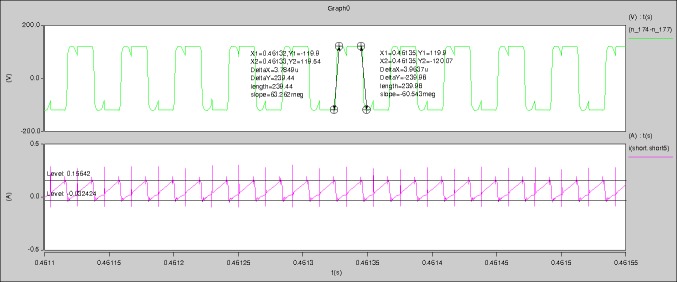
Waveforms under theoretical analysis.

**Fig 20:**
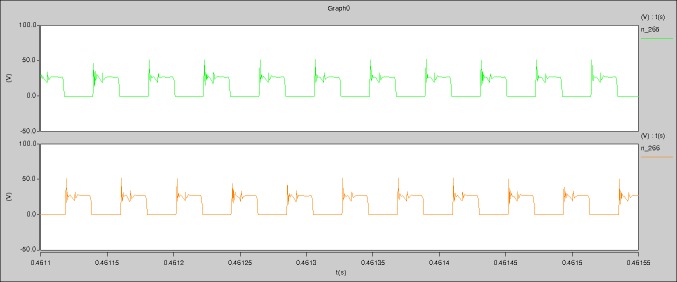
Waveforms under theoretical analysis.

**Fig 21:**
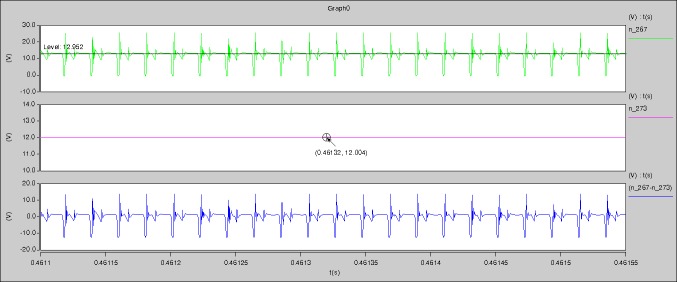
Waveforms under theoretical analysis.

**Fig 22:**
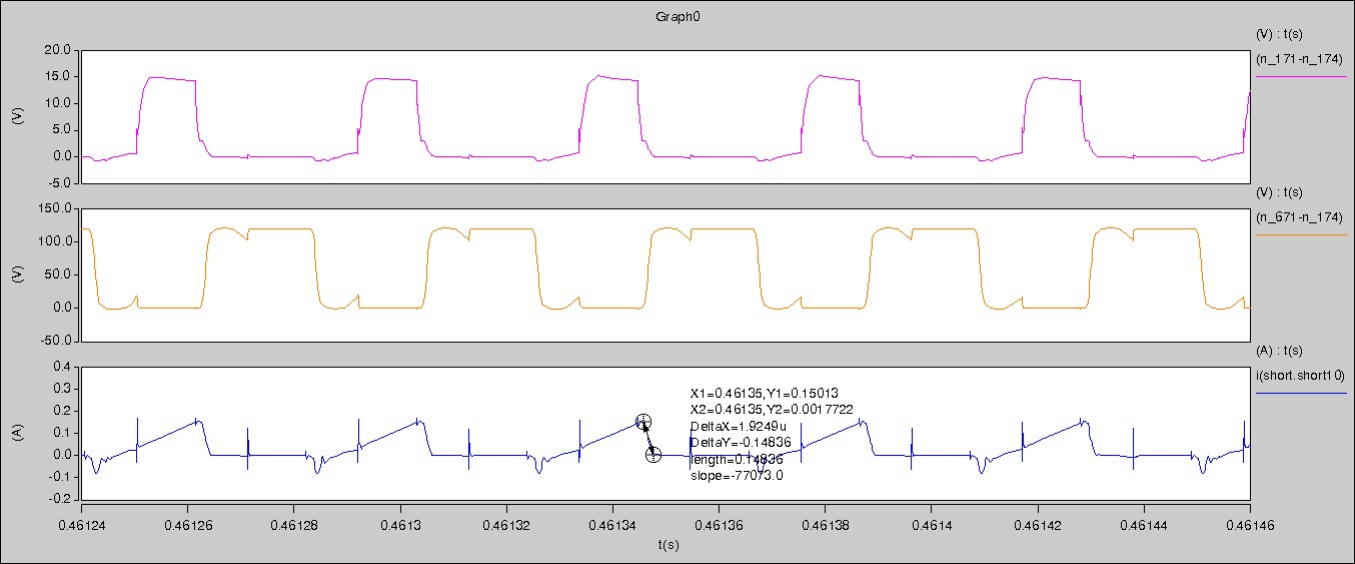
Waveforms under theoretical analysis.

The waveforms under theoretical analyses can be seen from Figs 18–22.

The meanings of Fig 18 from top to bottom are the gate-source voltage of Q1, the drain-source voltage of Q1, the gate-source voltage of Q4, and the drain-source voltage of Q4 respectively.

The meanings of Fig 19 from top to bottom are the midpoint voltage of two arms, and the input current respectively.

The meanings of Fig 20 from top to bottom are the reverse voltage of D1, and the reverse voltage of D2 respectively.

The meanings of Fig 21 from top to bottom are the total voltage corresponding to the secondary side of transformer and the Schottky rectifier diode, the output voltage, and the voltage of filter inductor respectively.

The meanings of Fig 22 from top to bottom are the gate-source voltage of Q1, the drain-source voltage of Q1, and the branch current of Q1 respectively.

Based on Figs 18–22, the following results can be obtained.

1) The drain-source voltage of Q1 and Q4 can be estimated as zero when they are turning-on.

2) The approximate amplitude of the midpoint voltage of two arms is 120.0V. And the approximate amplitude of the voltage of filter inductor is 1.5V.

3) The input current can be treated as triangular waveform and the rise time is close to the half of switching period *T*/2.

4) The average input current and the output voltage are 0.0620A and 12.004V.

5) The rise time and fall time of U_AB_ are 3.7849μs and 3.9637μs.

6) The fluctuation degree of the reverse bias voltage related to the D1 and D2 is large.

7) The conduction time of each MOSFET in each switching period t_on_ is 11μs.

**I_o_ = 1A**

**Fig 23:**
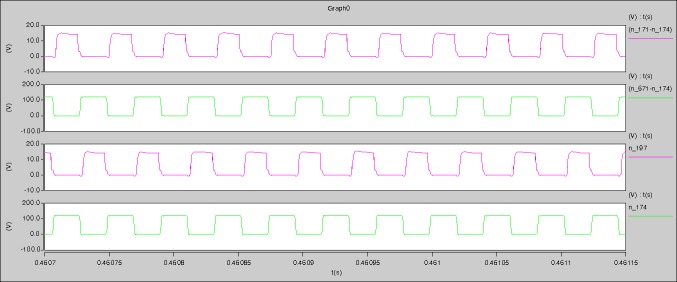
Waveforms under theoretical analysis.

**Fig 24:**
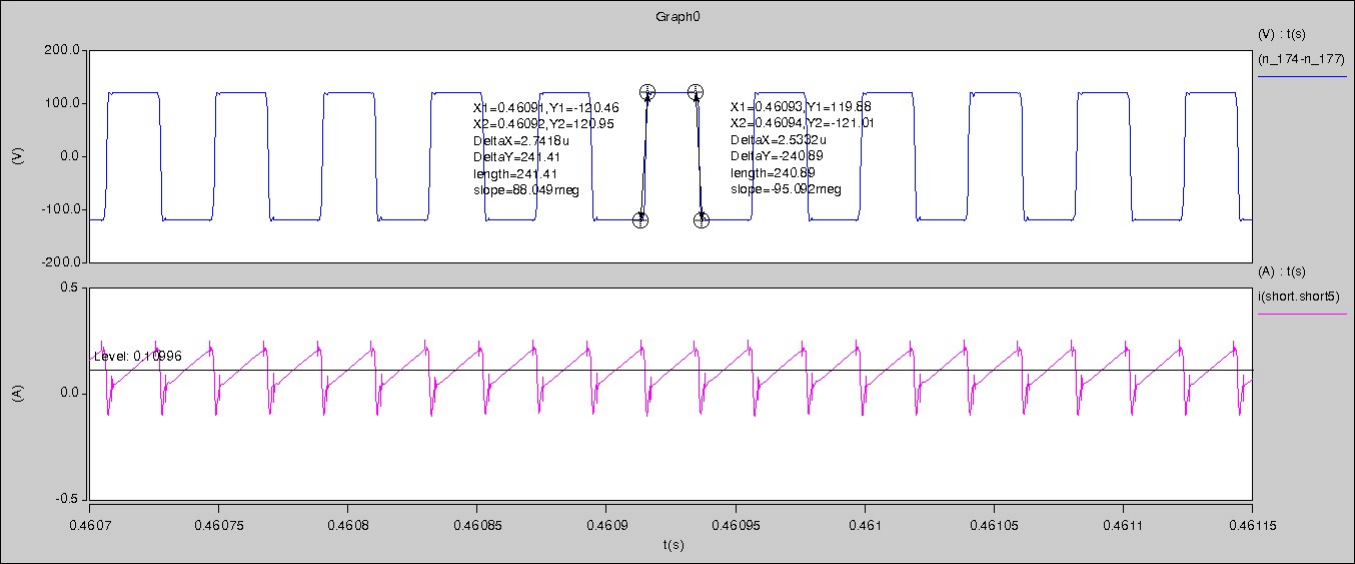
Waveforms under theoretical analysis.

**Fig 25:**
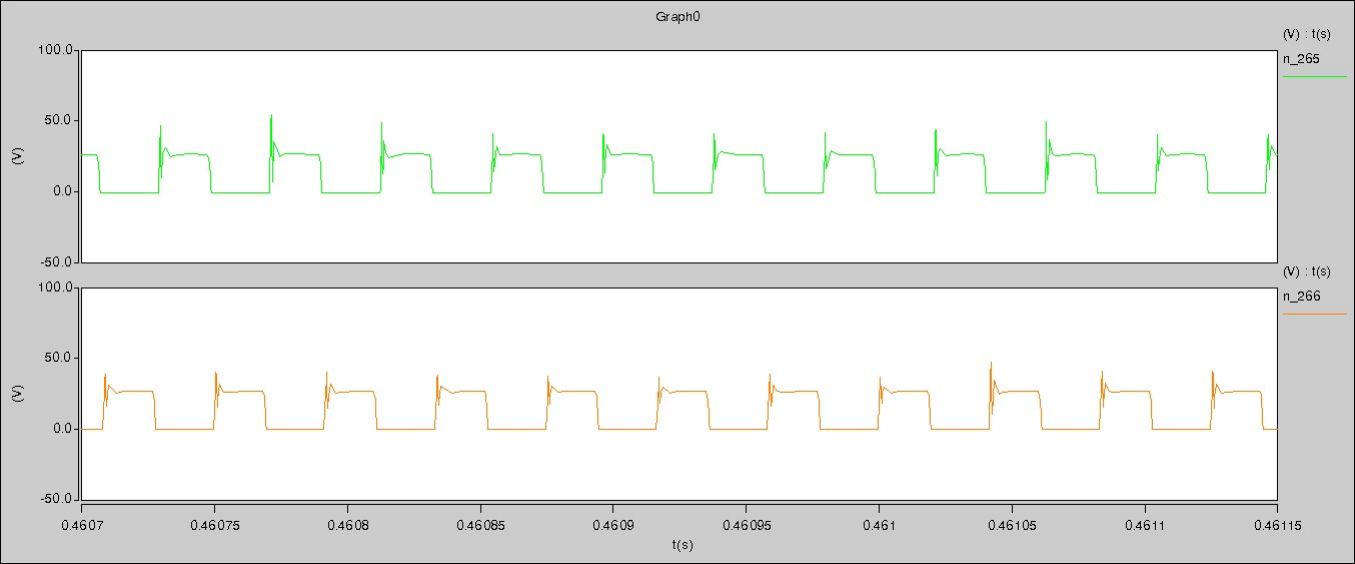
Waveforms under theoretical analysis.

**Fig 26:**
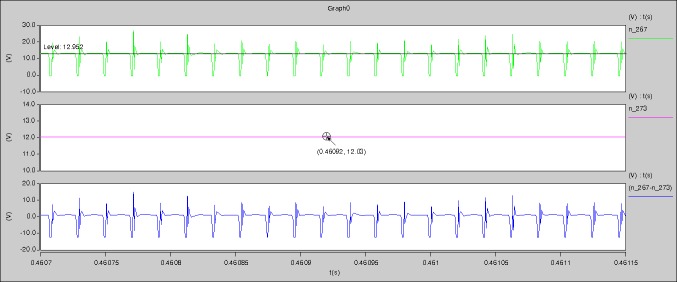
Waveforms under theoretical analysis.

**Fig 27:**
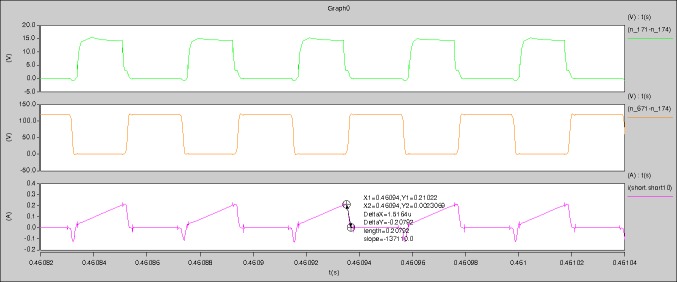
Waveforms under theoretical analysis.

The waveforms under theoretical analyses can be seen from Figs 23–27.

The meanings of Fig 23 from top to bottom are the gate-source voltage of Q1, the drain-source voltage of Q1, the gate-source voltage of Q4, and the drain-source voltage of Q4 respectively.

The meanings of Fig 24 from top to bottom are the midpoint voltage of two arms, and the input current respectively.

The meanings of Fig 25 from top to bottom are the reverse voltage of D1, and the reverse voltage of D2 respectively.

The meanings of Fig 26 from top to bottom are the total voltage corresponding to the secondary side of transformer and the Schottky rectifier diode, the output voltage, and the voltage of filter inductor respectively.

The meanings of Fig 27 from top to bottom are the gate-source voltage of Q1, the drain-source voltage of Q1, and the branch current of Q1 respectively.

Based on Figs 23–27, the following results can be obtained.

1) The drain-source voltage of Q1 and Q4 can be estimated as zero when they are turning-on.

2) The approximate amplitude of the midpoint voltage of two arms is 120.0V. And the approximate amplitude of the voltage of filter inductor is 1.5V.

3) The input current can be almost treated as triangular waveform and the rise time is close to the half of switching period *T*/2.

4) The average input current and the output voltage are 0.10996A and 12.030V.

5) The rise time and fall time of U_AB_ are 2.7418μs and 2.5332μs.

6) The fluctuation degree of the reverse bias voltage related to the D1 and D2 is large.

7) The conduction time of each MOSFET in each switching period t_on_ is 17μs.

**Fig 28:**
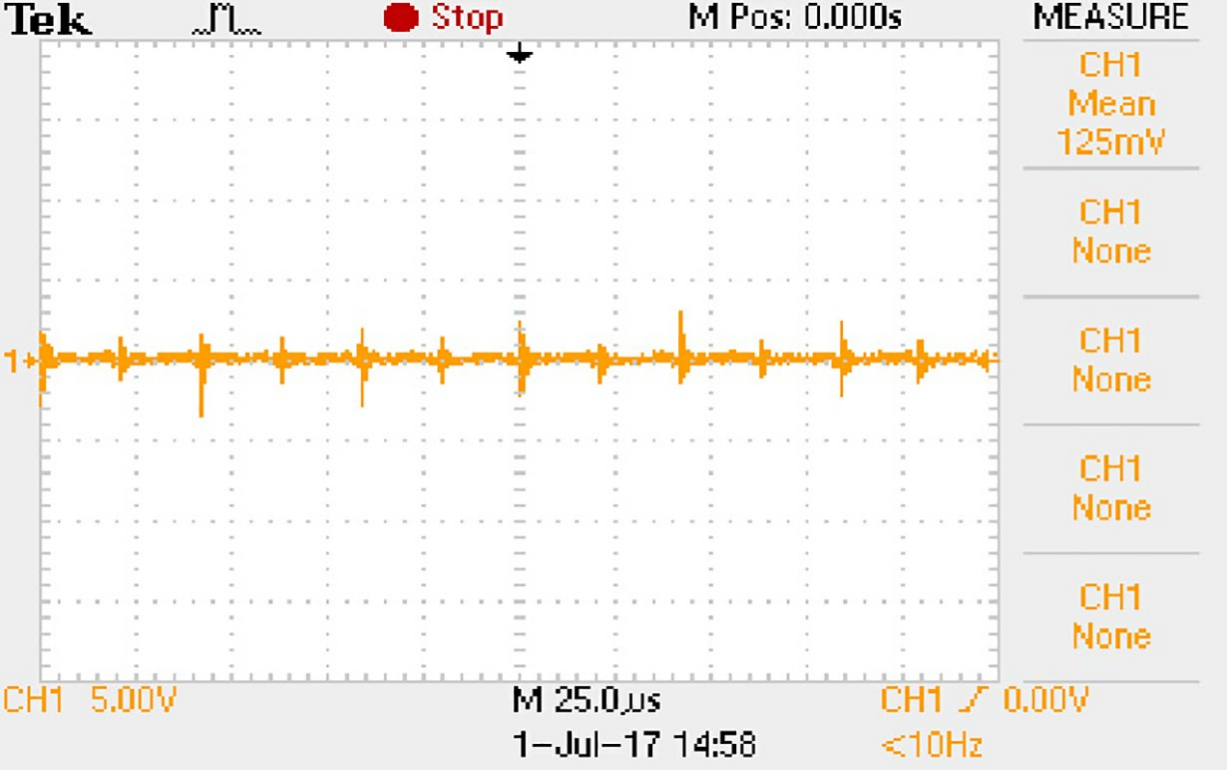
Actual measured input current.

In order to solve (6) and (8) in the proposed model, it is also necessary to obtain the actual average input current when the level of output current I_o_ is 1A. The experimental waveform of input current can be seen in Fig 28. It is the voltage waveform of 1Ω sampling resistor, so it can reflect the input current.

Based on Fig 28, the following results can be obtained.

1) The input current can be almost treated as triangular waveform and the rise time is close to the half of switching period *T*/2.

2) The average input current is 0.125A.

### 3.2 Proposed model

#### (A) Calculated parameters

Based on Fig 13, Fig 15, and Fig 17, it can be known that the *P*_ine(1)_, *P*_one(1)_, *P*_in1(1)_ and *P*_o1(1)_ in (3) are 2.415W, 1.2W, 1.9649W, and 1.2032W respectively. So the ΔP_core_ in (3) is 0.2267W.

Based on Fig 16, Fig 17, Fig 27, and Fig 28, it can be known that the *P*_ine(2)_, *P*_one(2)_, *P*_in1(2)_, and *P*_o1(2)_ are 15W, 12W, 13.1952W, and 12W respectively. When the *k*_1_ and *k*_2_ are calculated, *U*_off(1)_ and *U*_off(2)_ are estimated as 60V uniformly. *I*_off(1)_, *I*_off(2)_, *D*_off(1)_, and *D*_off(2)_ are calculated from the marks in Fig 16 and Fig 27 directly. So the *a* and *b* in (4) and (5) are 1.5870 and 0.1132 respectively.

In Fig 5 and Fig 6, the known default parameters are excluded and other parameters are as follows.

1) Eq (9) is solved according to the actual parameters of winding. These parameters can be seen in the appendix. We can know that R_P_, R_S1_, R_S2_, and R_Lf_ are 0.4982Ω, 0.0254Ω, 0.0254Ω, and 0.0348Ω respectively.

2) Eq (6) is applied. Then it can be known that R_core_ is 13.415kΩ when U_in_ is 115V and R_core_ is 13.126 kΩ when U_in_ is 120V.

3) The undetermined parameters of SPICE model related to the D1 and D2 are listed as follows:

$$Is=451.69nA,\;N=1,\;V_{T}=26mV,\;C_{j0}=0nF,\;V_{j}=1V,\;and\;M=0.64.$$

4) The ESR of filter capacitor C is 0.1Ω.

5) R_switch_ in Fig 6 is determined by comprehending the Fig 5, the (7), and the (8).

#### (B) Presentation of theoretical results

According to the Fig 5, the Fig 6, and the aforementioned parameters, the proposed model is established in the Saber software. When the levels of output current I_o_ are 0.1A, 0.5A, and 1A respectively, the following waveforms can be seen.

**I_o_ = 0.1A**

**Fig 29:**
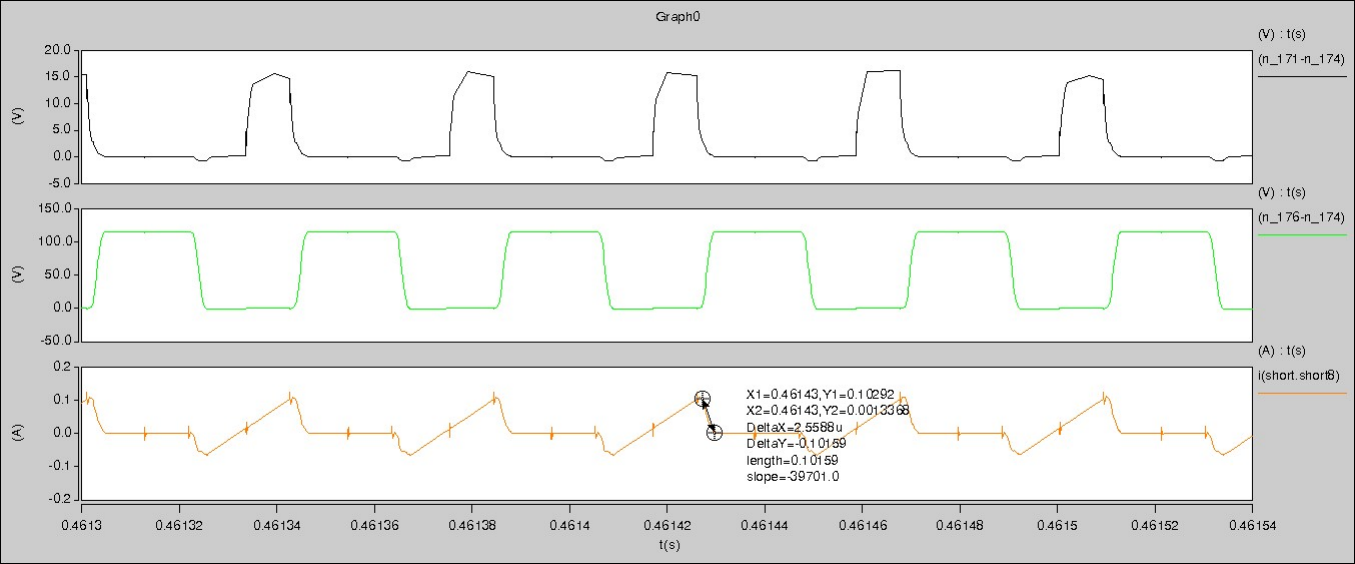
Waveforms under theoretical analysis.

**Fig 30:**
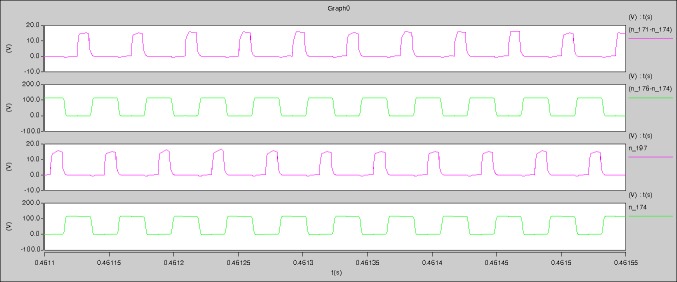
Waveforms under theoretical analysis.

**Fig 31:**
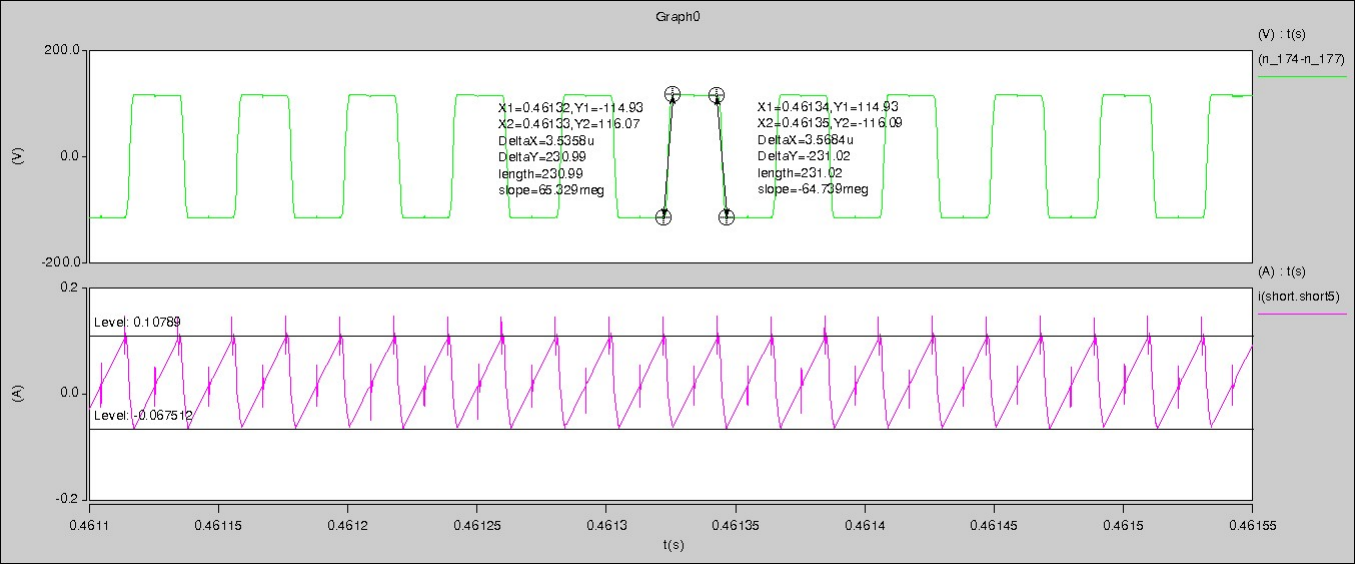
Waveforms under theoretical analysis.

**Fig 32:**
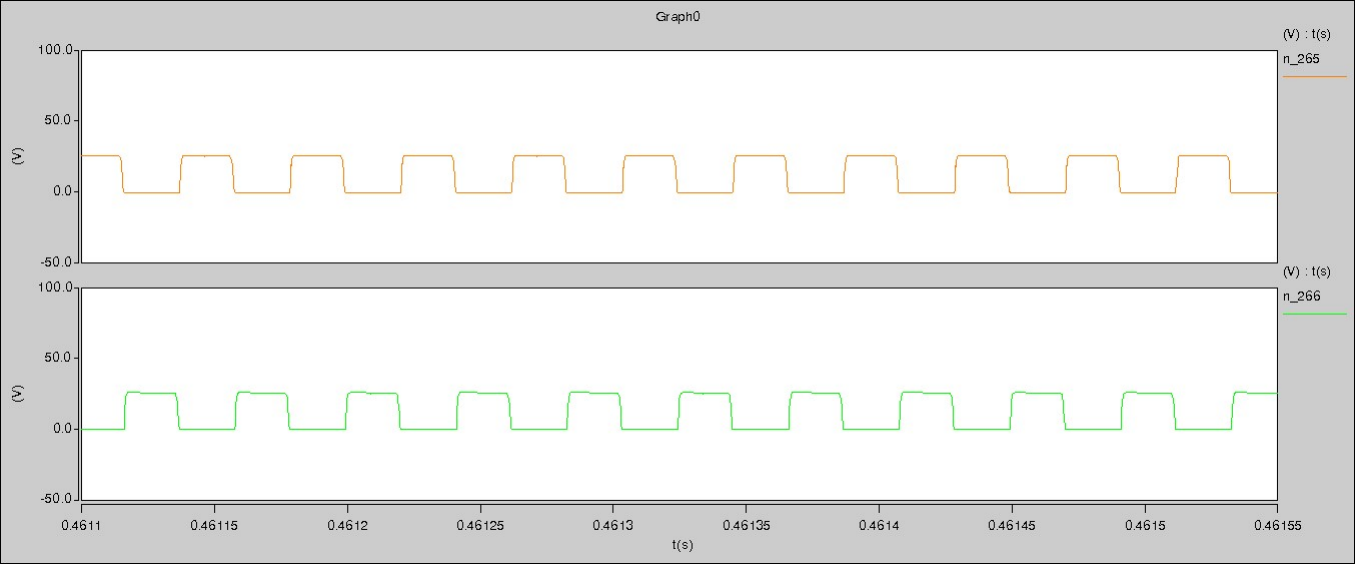
Waveforms under theoretical analysis.

**Fig 33:**
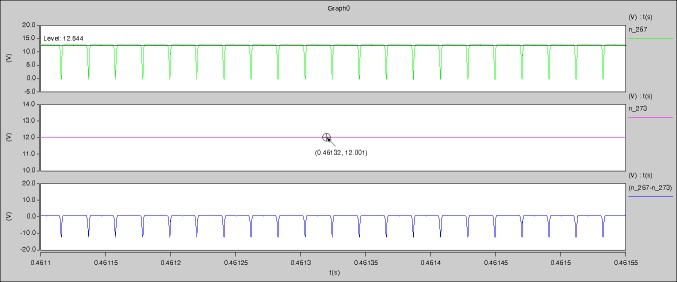
Waveforms under theoretical analysis.

Fig 29 can be obtained by aided analyses on Fig 5. R_switch_ is equal to 63.308kΩ according to the Fig 29, the (7), and the (8), so Figs 30–33 can be obtained on the bases of Fig 6. When (7) and (8) are operated, *U*_off(Io)_ is estimated as 60V approximately. *I*_off(Io)_ and *D*_off(Io)_ are calculated from the marks in Fig 29 directly. They are 0.0521A and 6.1411% respectively.

The waveforms under theoretical analyses can be seen from Figs 29–33.

The meanings of Fig 29 from top to bottom are the gate-source voltage of Q1, the drain-source voltage of Q1, and the branch current of Q1 respectively.

The meanings of Fig 30 from top to bottom are the gate-source voltage of Q1, the drain-source voltage of Q1, the gate-source voltage of Q4, and the drain-source voltage of Q4 respectively.

The meanings of Fig 31 from top to bottom are the midpoint voltage of two arms, and the input current respectively.

The meanings of Fig 32 from top to bottom are the reverse voltage of D1, and the reverse voltage of D2 respectively.

The meanings of Fig 33 from top to bottom are the total voltage corresponding to the secondary side of transformer and the Schottky rectifier diode, the output voltage, and the voltage of filter inductor respectively.

Based on Figs 29–33, the following results can be obtained.

1) The drain-source voltage of Q1 and Q4 can be estimated as zero when they are turning-on.

2) The approximate amplitude of the midpoint voltage of two arms is 115.0V. And the approximate amplitude of the voltage of filter inductor is 1.0V.

3) The input current can be treated as triangular waveform and the rise time is close to the half of switching period *T*/2.

4) The average input current and the output voltage are 0.0202A and 12.001V.

5) The rise time and fall time of U_AB_ are 3.5358μs and 3.5684μs.

6) The fluctuation degree of the reverse bias voltage related to the D1 and D2 is small.

7) The conduction time of each MOSFET in each switching period t_on_ is 9μs.

**I_o_ = 0.5A**

**Fig 34:**
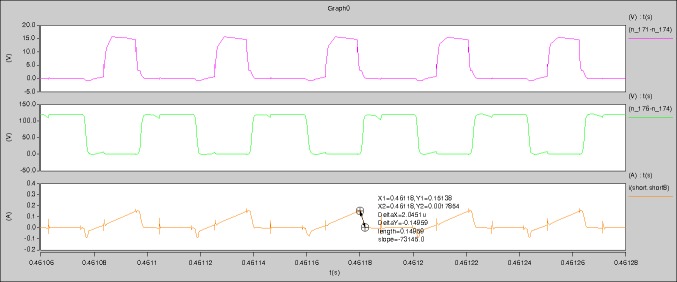
Waveforms under theoretical analysis.

**Fig 35:**
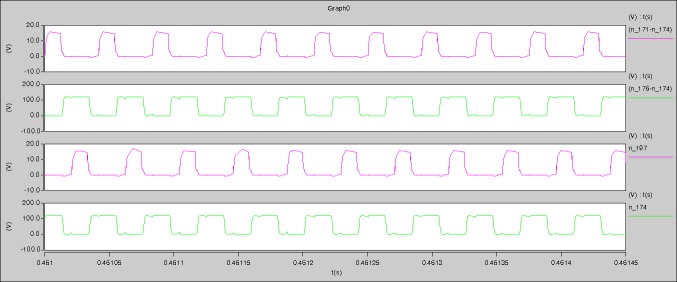
Waveforms under theoretical analysis.

**Fig 36:**
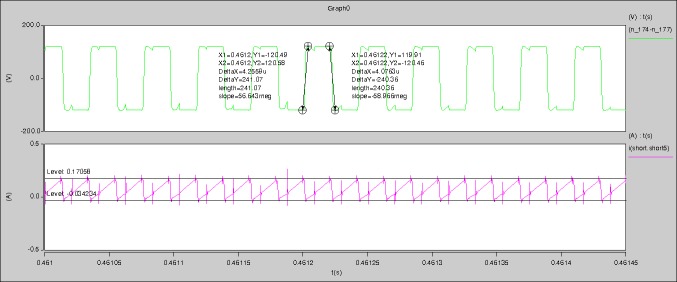
Waveforms under theoretical analysis.

**Fig 37:**
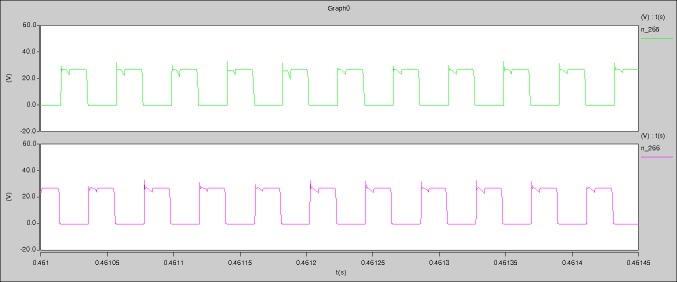
Waveforms under theoretical analysis.

**Fig 38:**
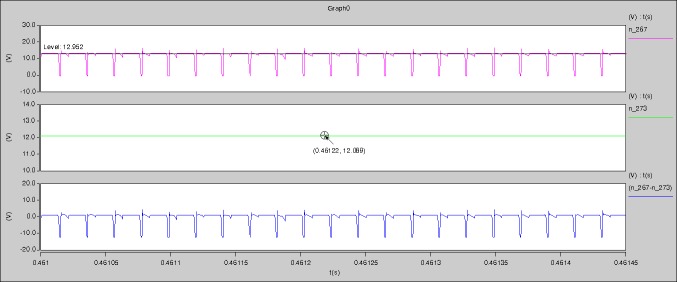
Waveforms under theoretical analysis.

Fig 34 is the results of Fig 5. R_switch_ is equal to 17.606kΩ according to the Fig 34, the (7), and the (8), so Figs 35–38 can be obtained on the bases of Fig 6. When the (7) and the (8) are operated, *U*_off(Io)_ is estimated as 60V. *I*_off(Io)_ and *D*_off(Io)_ are calculated from the marks in Fig 34 directly. They are 0.0766A and 4.9082% respectively.

The waveforms under theoretical analyses can be seen from Figs 34–38.

The meanings of Fig 34 from top to bottom are the gate-source voltage of Q1, the drain-source voltage of Q1, and the branch current of Q1 respectively.

The meanings of Fig 35 from top to bottom are the gate-source voltage of Q1, the drain-source voltage of Q1, the gate-source voltage of Q4, and the drain-source voltage of Q4 respectively.

The meanings of Fig 36 from top to bottom are the midpoint voltage of two arms, and the input current respectively.

The meanings of Fig 37 from top to bottom are the reverse voltage of D1, and the reverse voltage of D2 respectively.

The meanings of Fig 38 from top to bottom are the total voltage corresponding to the secondary side of transformer and the Schottky rectifier diode, the output voltage, and the voltage of filter inductor respectively.

Based on Figs 34–38, the following results can be obtained.

1) The drain-source voltage of Q1 and Q4 can be estimated as zero when they are turning-on.

2) The approximate amplitude of the midpoint voltage of two arms is 120.0V. And the approximate amplitude of the voltage of filter inductor is 1.5V.

3) The input current can be treated as triangular waveform and the rise time is close to the half of switching period *T*/2.

4) The average input current and the output voltage are 0.0682A and 12.089V.

5) The rise time and fall time of U_AB_ are 4.2559μs and 4.0763μs.

6) The fluctuation degree of the reverse bias voltage related to the D1 and D2 is small.

7) The conduction time of each MOSFET in each switching period t_on_ is 12μs.

**I_o_ = 1A**

**Fig 39:**
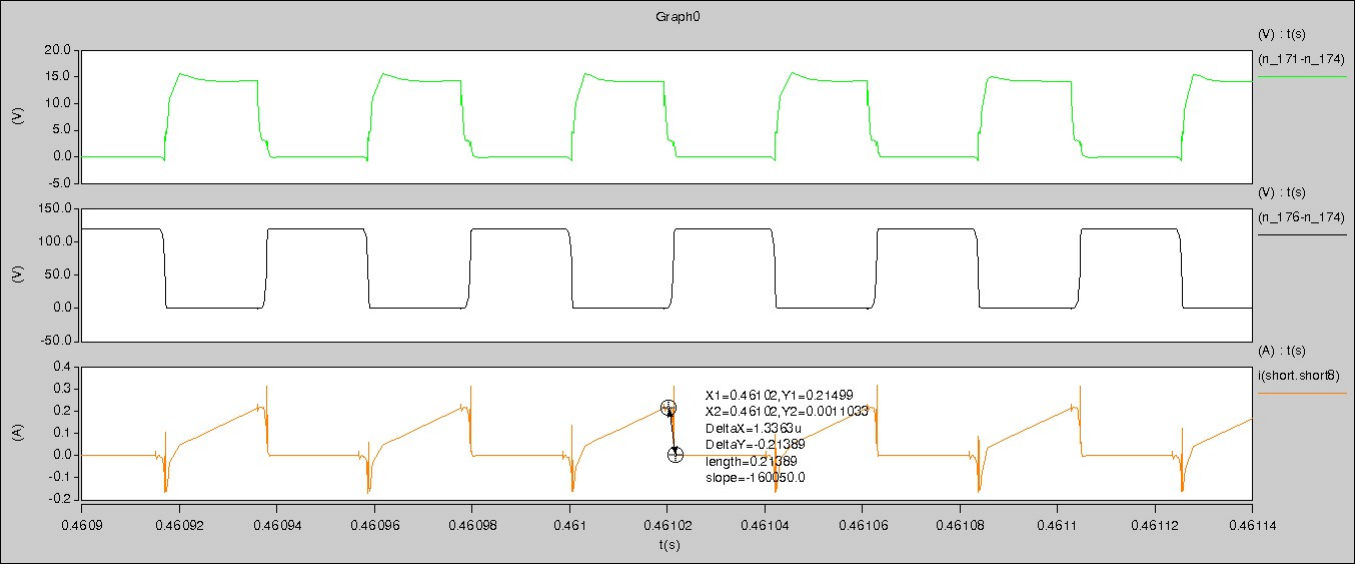
Waveforms under theoretical analysis.

**Fig 40:**
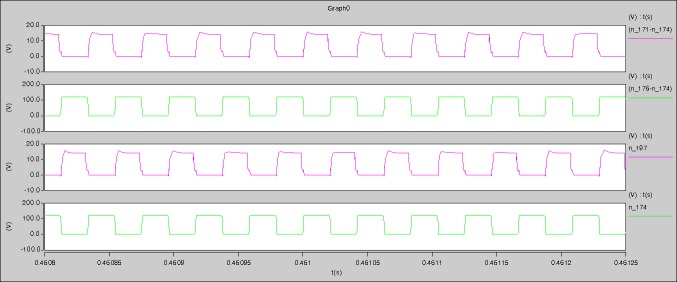
Waveforms under theoretical analysis.

**Fig 41:**
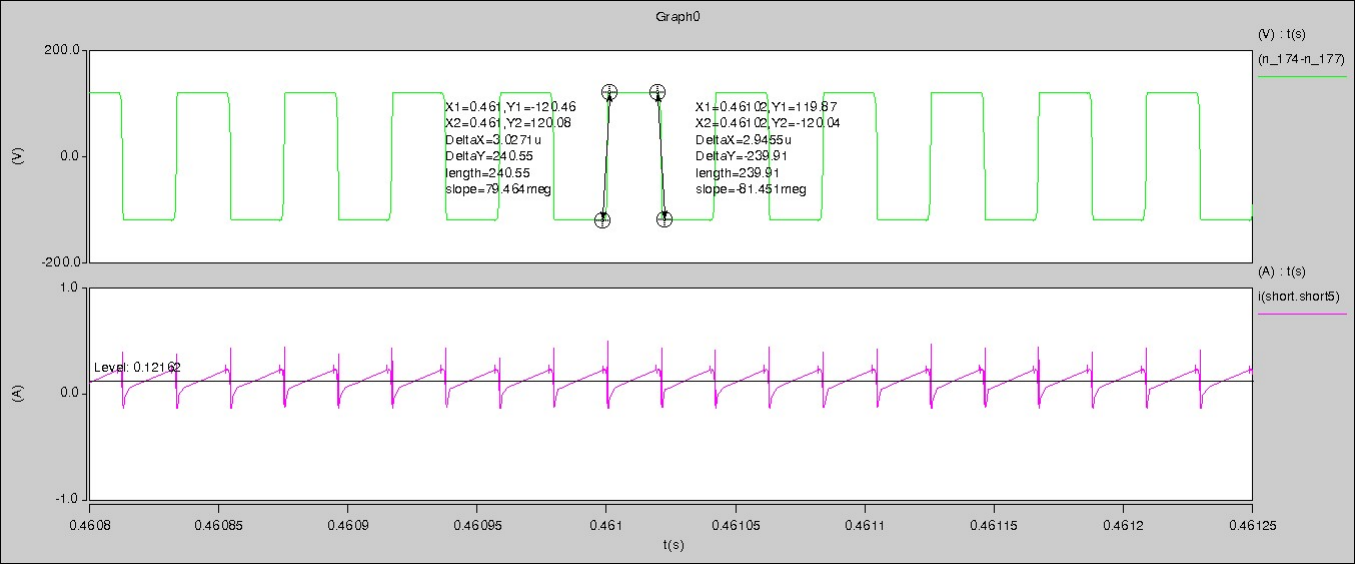
Waveforms under theoretical analysis.

**Fig 42:**
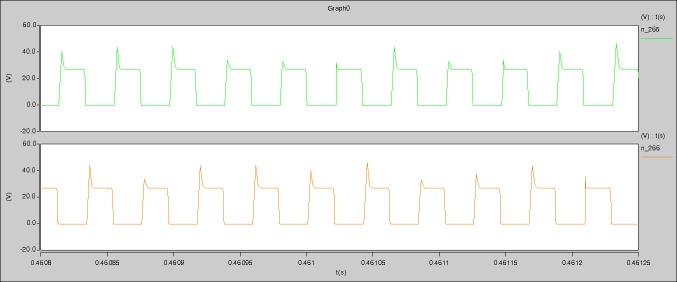
Waveforms under theoretical analysis.

**Fig 43:**
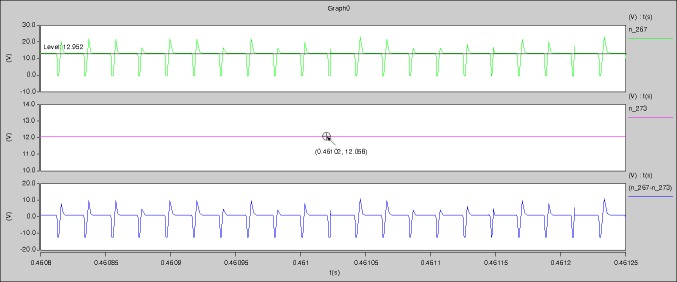
Waveforms under theoretical analysis.

Fig 39 is the results of Fig 5. R_switch_ is equal to 10.185kΩ according to Fig 39, the (7), and the (8), so Figs 40–43 can be obtained on the bases of Fig 6. When the (7) and the (8) are operated, *U*_off(Io)_ is estimated as 60V. *I*_off(Io)_ and *D*_off(Io)_ are calculated from the marks in Fig 39 directly. They are 0.1080A and 3.2071% respectively.

The waveforms under theoretical analyses can be seen from Figs 39–43.

The meanings of Fig 39 from top to bottom are the gate-source voltage of Q1, the drain-source voltage of Q1, and the branch current of Q1 respectively.

The meanings of Fig 40 from top to bottom are the gate-source voltage of Q1, the drain-source voltage of Q1, the gate-source voltage of Q4, and the drain-source voltage of Q4 respectively.

The meanings of Fig 41 from top to bottom are the midpoint voltage of two arms, and the input current respectively.

The meanings of Fig 42 from top to bottom are the reverse voltage of D1, and the reverse voltage of D2 respectively.

The meanings of Fig 43 from top to bottom are the total voltage corresponding to the secondary side of transformer and the Schottky rectifier diode, the output voltage, and the voltage of filter inductor respectively.

Based on Figs 39–43, the following results can be obtained.

1) The drain-source voltage of Q1 and Q4 can be estimated as zero when they are turning-on.

2) The approximate amplitude of the midpoint voltage of two arms is 120.0V. And the approximate amplitude of the voltage of filter inductor is 1.5V.

3) The input current can be almost treated as triangular waveform and the rise time is close to the half of switching period *T*/2.

4) The average input current and the output voltage are 0.12162A and 12.058V.

5) The rise time and fall time of U_AB_ are 3.0271μs and 2.9455μs.

6) The fluctuation degree of the reverse bias voltage related to the D1 and D2 is small.

7) The conduction time of each MOSFET in each switching period t_on_ is 19μs.

## 4 Experimental verification

Experimental platform for the isolated full-bridge DC-DC converter has been built so that the approximation degree between the two models and the actual converter can be verified. This platform is regulated by the real-time data acquisition and the control card Qs1501 which supports the real-time window target (RTWT) and the XPC target environment. The parameters of driving circuit and the main circuit have been described in the second section. They are listed in Table 1 as follows.

**Table 1:** Parameters of converter.

Input voltage U_in_	Case 1: 115V; Case 2: 120V
Output voltage U_o_	12V
Current under full load	10A
MOSFET	IRFP460
Antiparallel freewheeling diode	MUR3060WT
Leakage inductance	600μH
Excitation inductance	11.5mH
Turns ratio	8.42:1:1
Rectifier diode	V50100PW
Filter inductor	250μH
Filter capacitor	470μF
Switching frequency	24kHz
Driven resistors R_11_to R_14_	6.2Ω
Driven resistors R_21_ to R_24_	10kΩ
Zener diodes V_z1_ to V_z4_	IN4744A

When the levels of output current I_o_ are 0.1A, 0.5A, and 1A respectively, the following waveforms can be seen.

**I_o_ = 0.1A**

**Fig 44:**
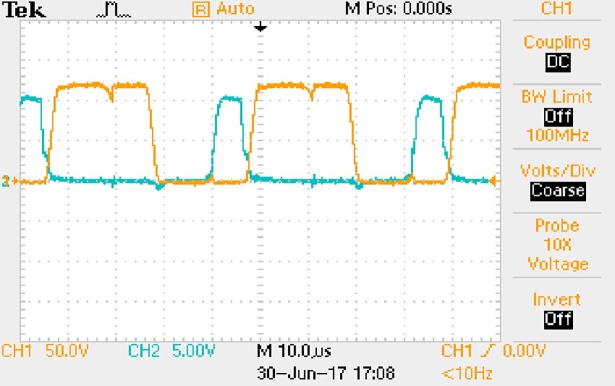
Measured waveforms.

**Fig 45:**
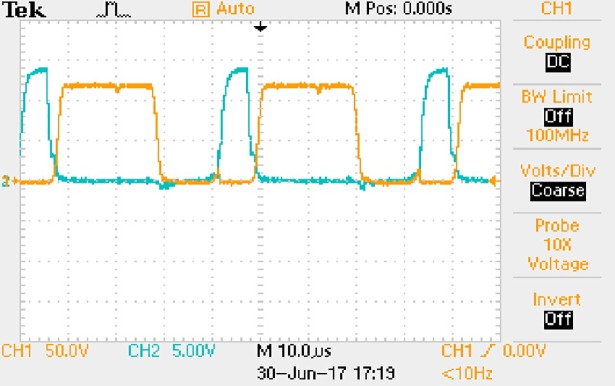
Measured waveforms.

**Fig 46:**
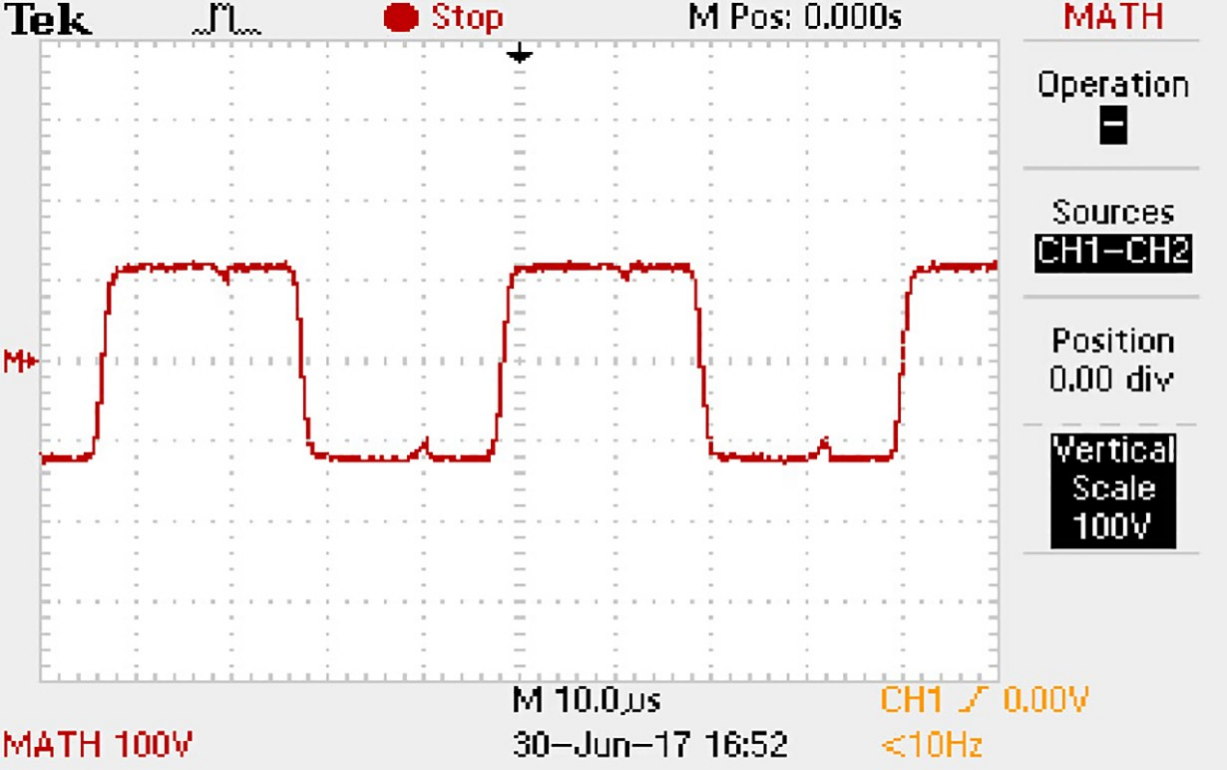
Measured waveforms.

**Fig 47:**
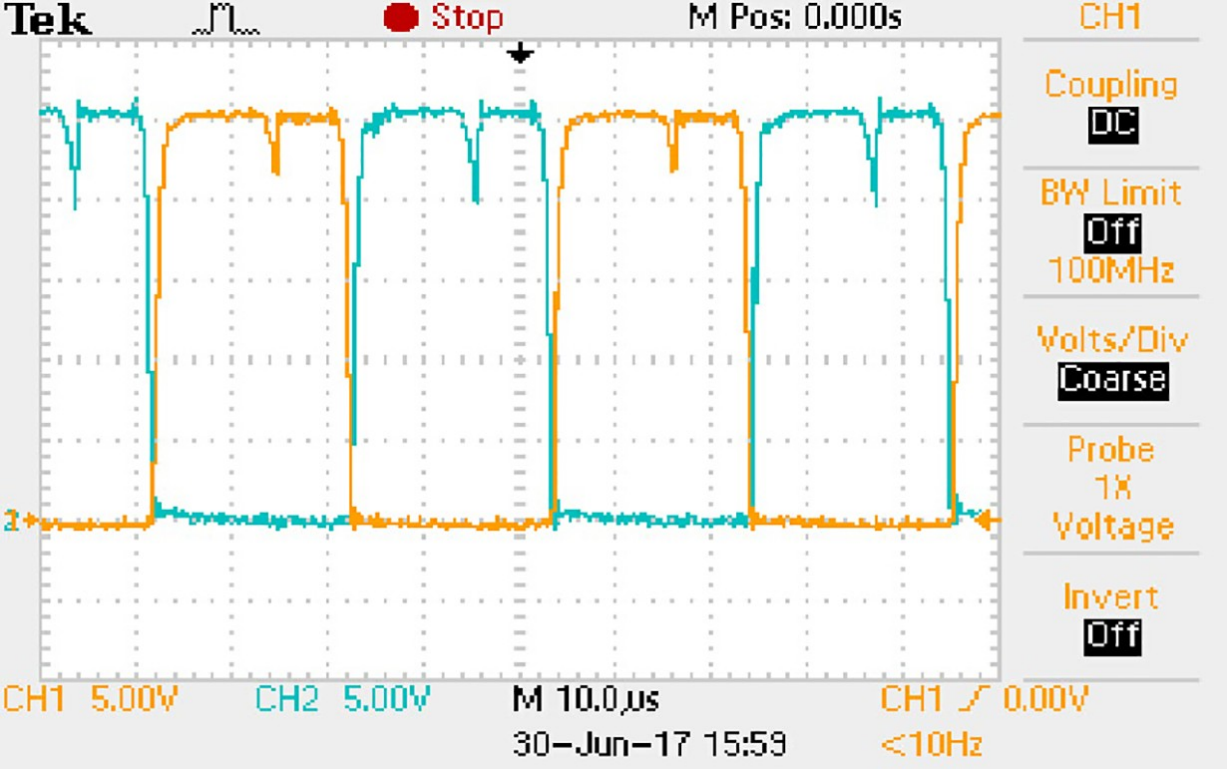
Measured waveforms.

**Fig 48:**
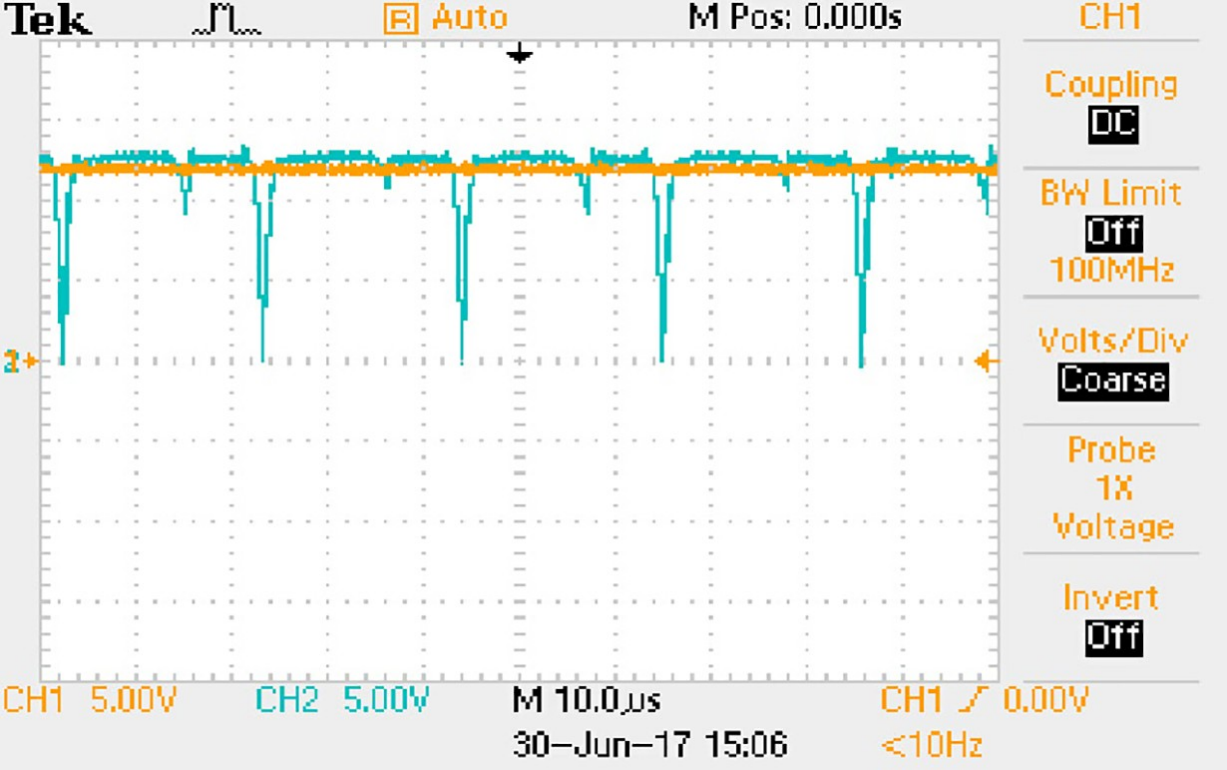
Measured waveforms.

**Fig 49:**
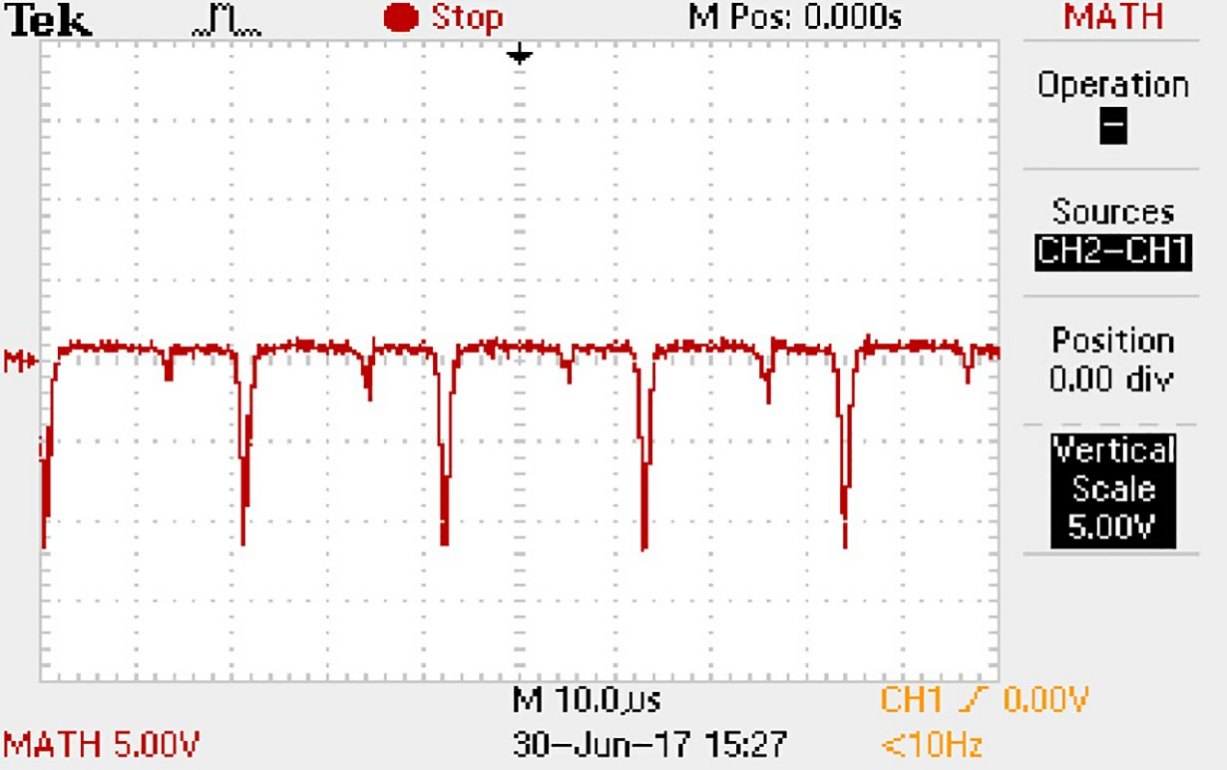
Measured waveforms.

The measured waveforms can be seen from Figs 44–49.

In Fig 44, the CH1 means the drain-source voltage of Q1 and the CH2 means the gate-source voltage of Q1.

In Fig 45, the CH1 means the drain-source voltage of Q4 and the CH2 means the gate-source voltage of Q4.

In Fig 46, the waveform means the midpoint voltage of two arms.

In Fig 47, the CH1 means the reverse voltage of D2 and the CH2 means the reverse voltage of D1.

In Fig 48, the CH1 means the output voltage and the CH2 means the total voltage corresponding to the secondary side of transformer and the Schottky rectifier diode.

In Fig 49, the waveform means the voltage of filter inductor.

Based on Figs 44–49, the following results can be obtained.

1) The drain-source voltage of Q1 and Q4 can be estimated as zero when they are turning-on.

2) The approximate amplitude of the midpoint voltage of two arms is 115.0V. And the approximate amplitude of the voltage of filter inductor is 0.8V.

3) The output voltage is 12.0V.

4) The rise time and fall time of U_AB_ are both 3μs.

5) The fluctuation degree of the reverse bias voltage related to the D1 and D2 is small.

6) The conduction time of each MOSFET in each switching period t_on_ is 8μs.

**I_o_ = 0.5A**

**Fig 50:**
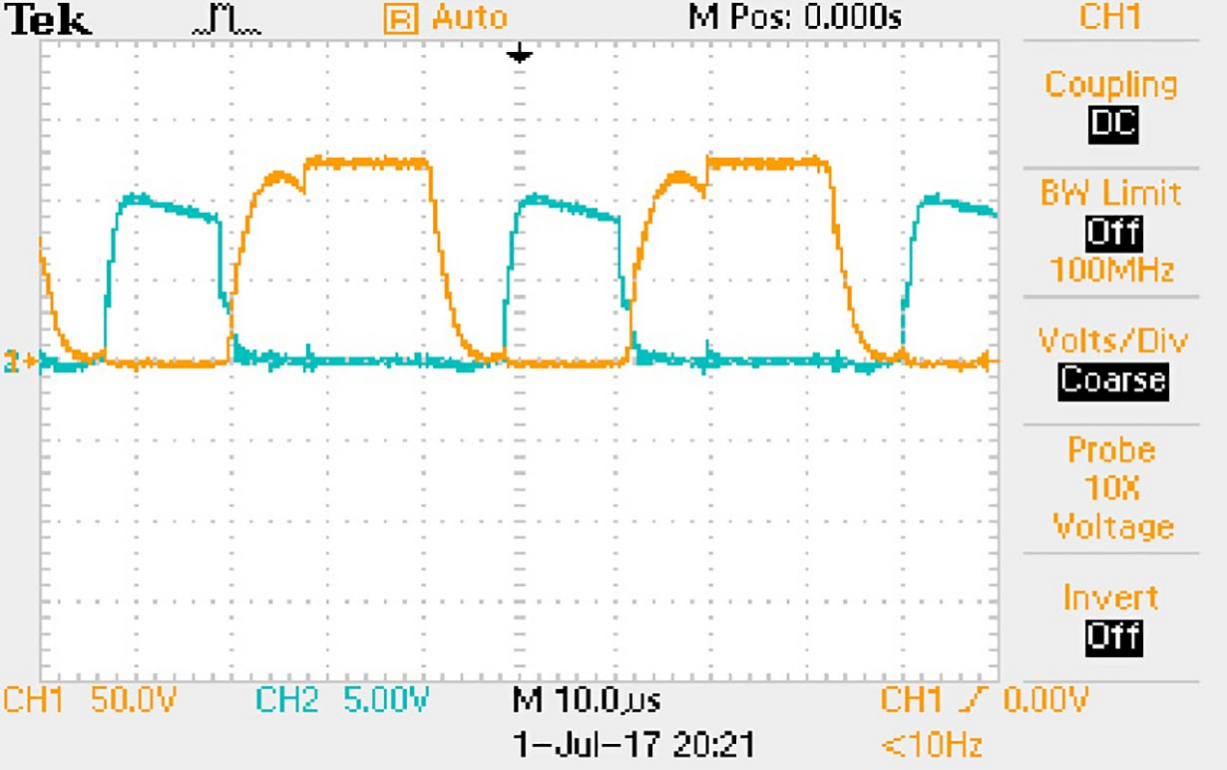
Measured waveforms.

**Fig 51:**
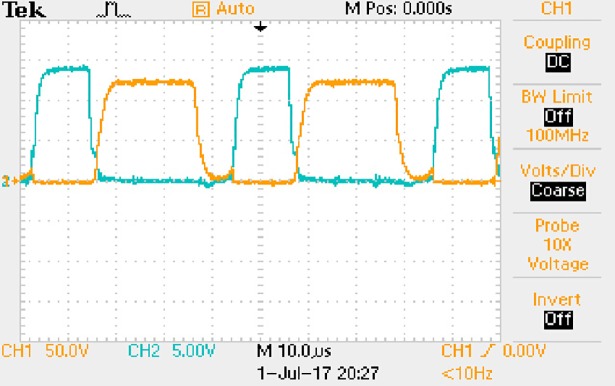
Measured waveforms.

**Fig 52:**
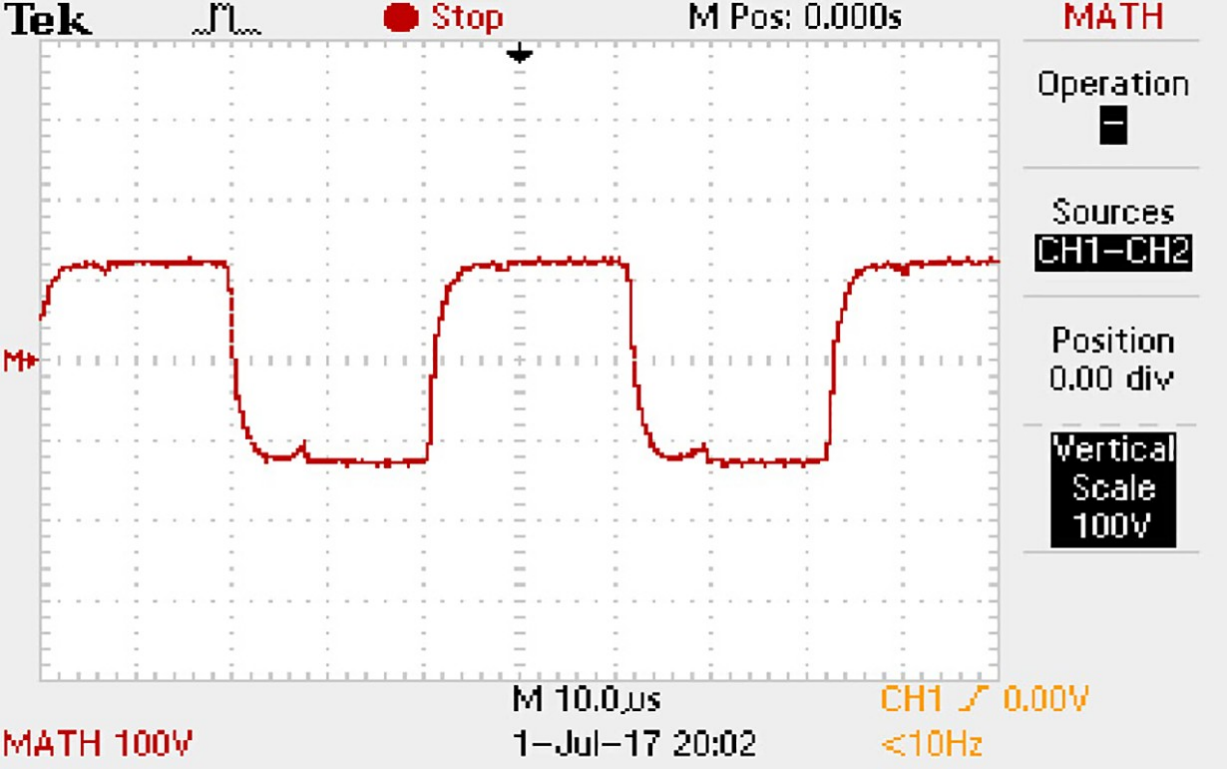
Measured waveforms.

**Fig 53:**
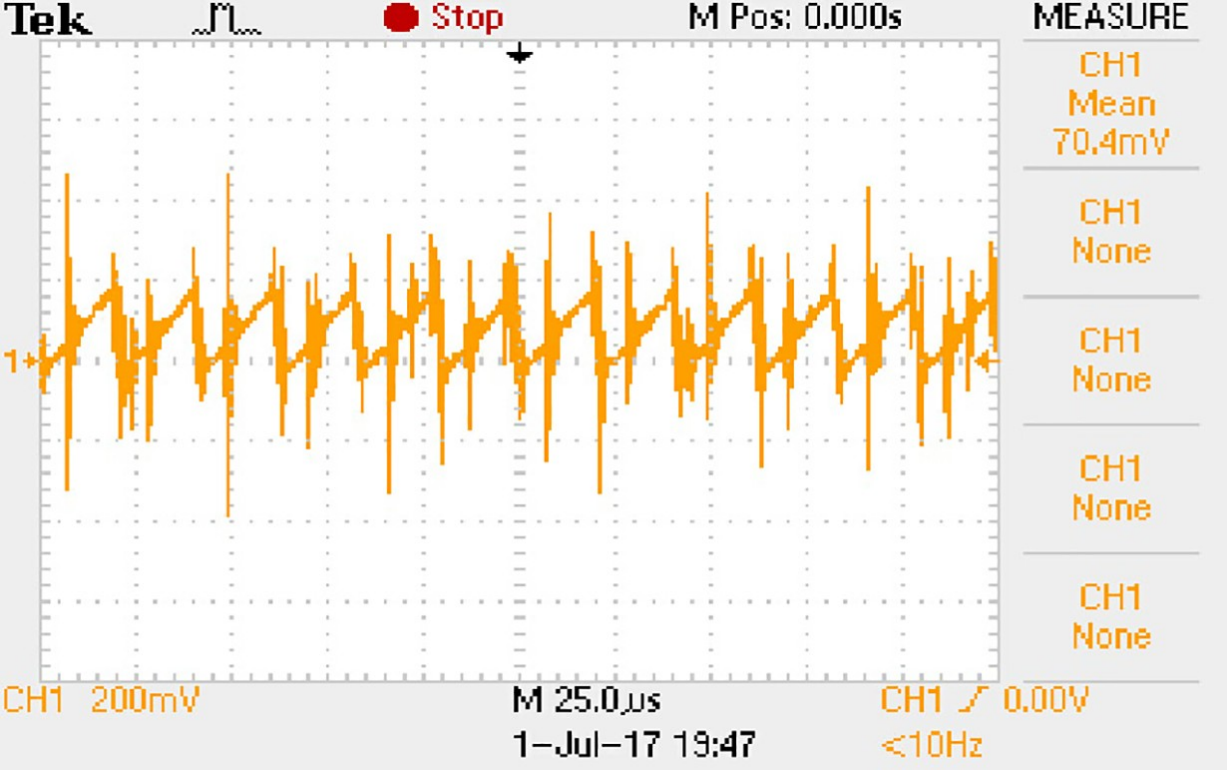
Measured waveforms.

**Fig 54:**
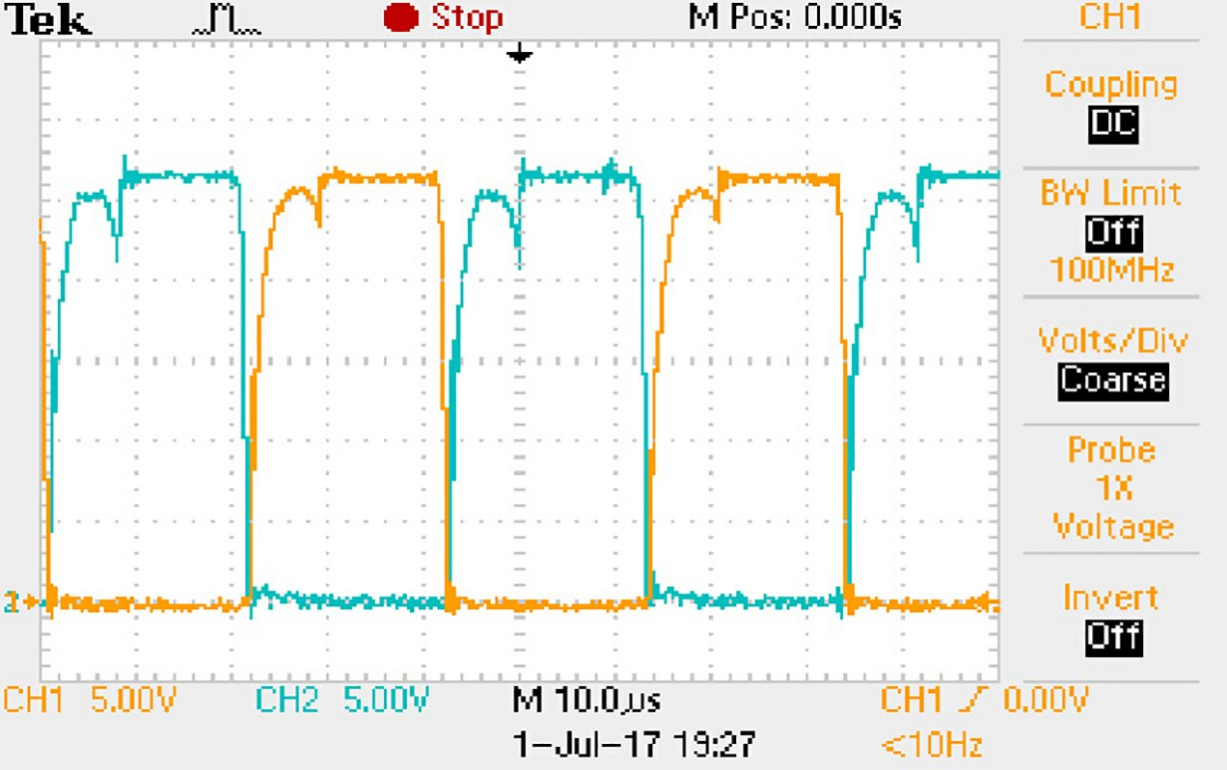
Measured waveforms.

**Fig 55:**
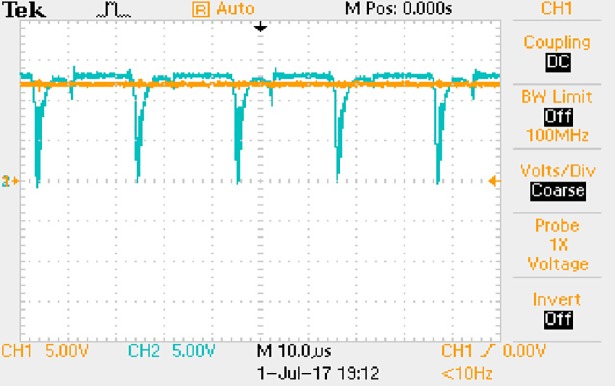
Measured waveforms.

**Fig 56:**
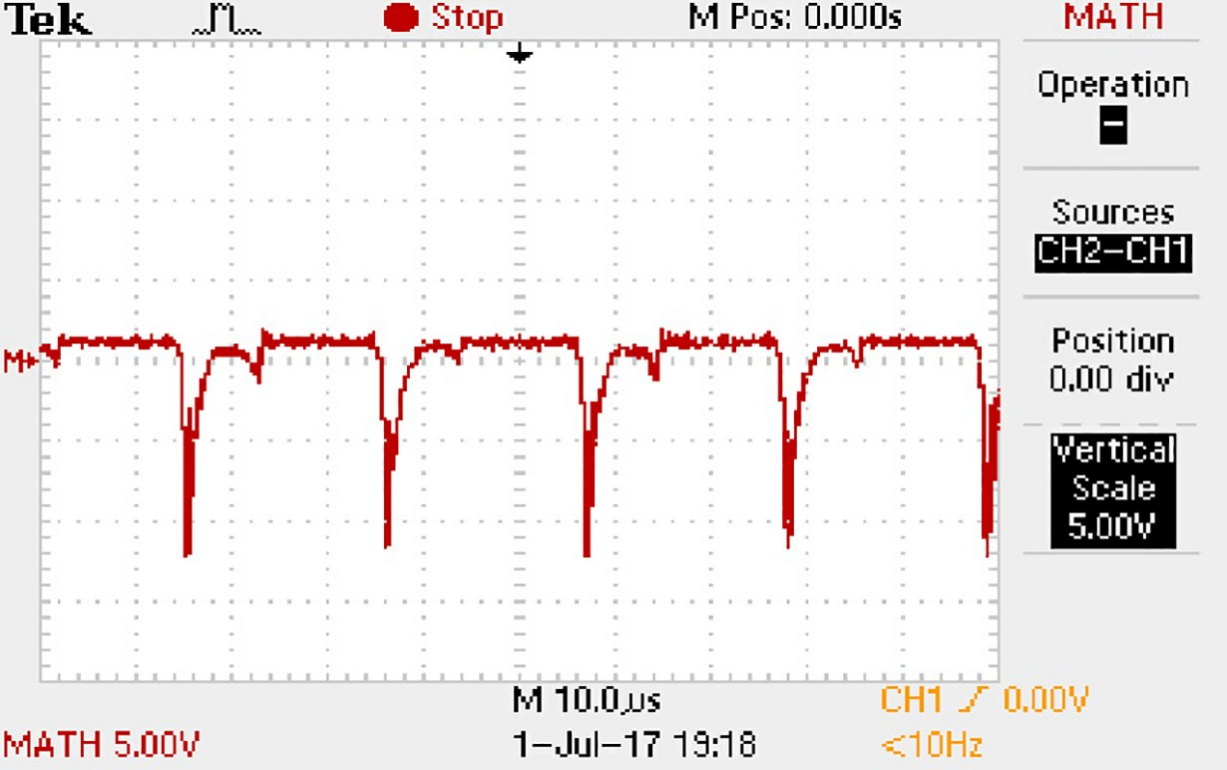
Measured waveforms.

The measured waveforms can be seen from Figs 50–56.

In Fig 50, the CH1 means the drain-source voltage of Q1 and the CH2 means the gate-source voltage of Q1.

In Fig 51, the CH1 means the drain-source voltage of Q4 and the CH2 means the gate-source voltage of Q4.

In Fig 52, the waveform means the midpoint voltage of two arms.

In Fig 53, the waveform means the input current obtained by the 1Ω sampling resistor.

In Fig 54, the CH1 means the reverse voltage of D2 and the CH2 means the reverse voltage of D1.

In Fig 55, the CH1 means the output voltage and the CH2 means the total voltage corresponding to the secondary side of transformer and the Schottky rectifier diode.

In Fig 56, the waveform means the voltage of filter inductor.

Based on Figs 50–56, the following results can be obtained.

1) The drain-source voltage of Q1 and Q4 can be estimated as zero when they are turning-on.

2) The approximate amplitude of the midpoint voltage of two arms is 120.0V. And the approximate amplitude of the voltage of filter inductor is 1.2V.

3) The input current can be treated as triangular waveform and the rise time is close to the half of switching period *T*/2.

4) The average input current and the output voltage are 0.0704A and 12.0V.

5) The rise time and fall time of U_AB_ are both 5μs.

6) The fluctuation degree of the reverse bias voltage related to the D1 and D2 is small.

7) The conduction time of each MOSFET in each switching period t_on_ is 12μs.

**I_o_ = 1A**

**Fig 57:**
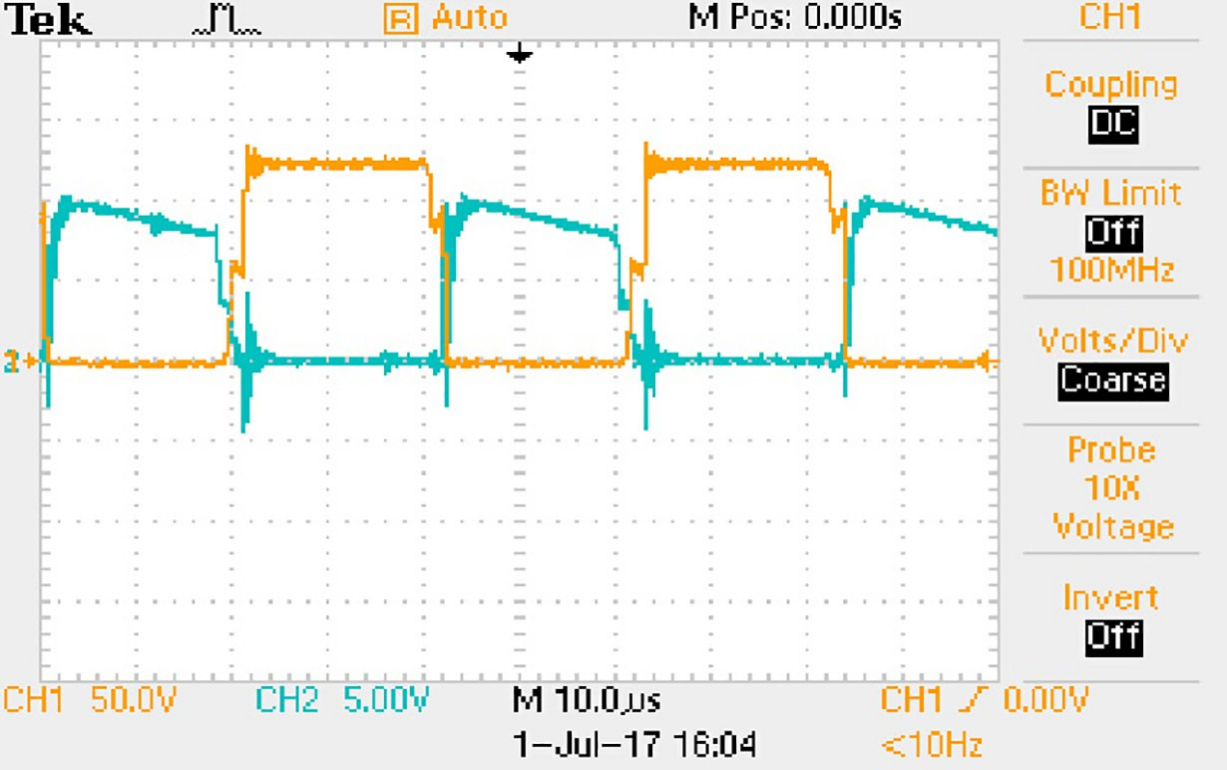
Measured waveforms.

**Fig 58:**
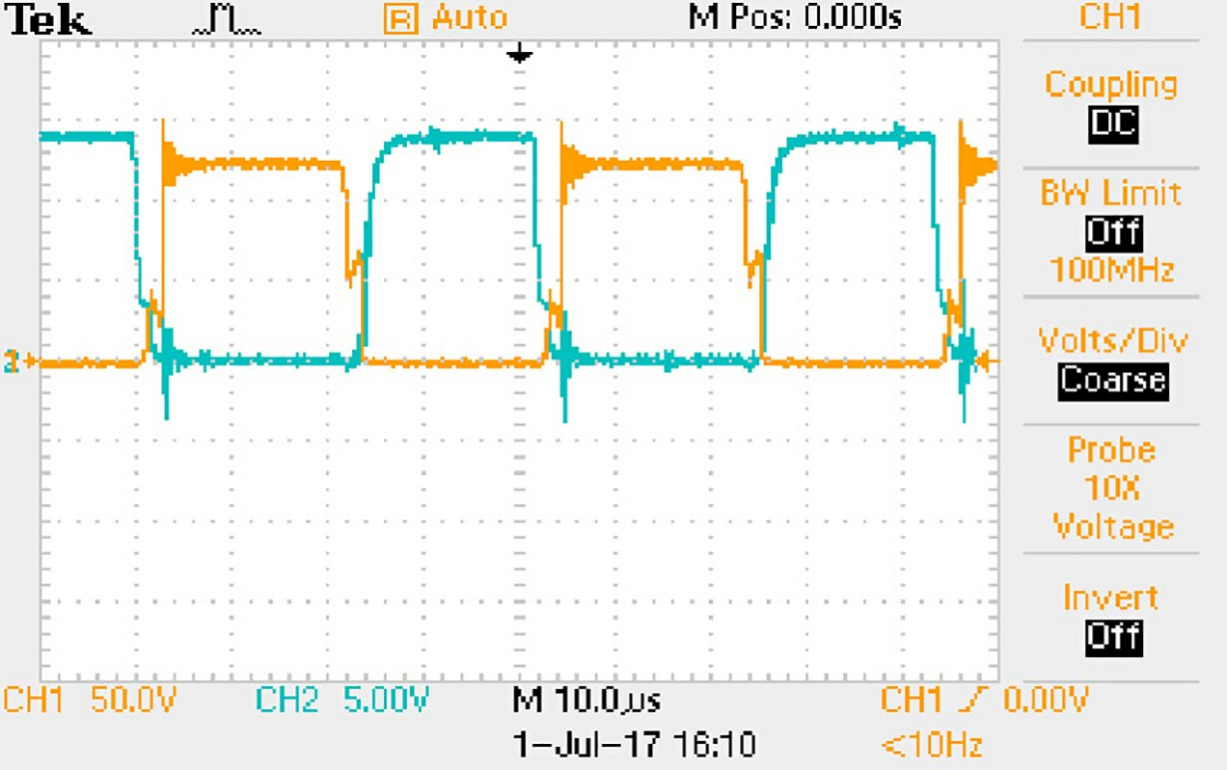
Measured waveforms.

**Fig 59:**
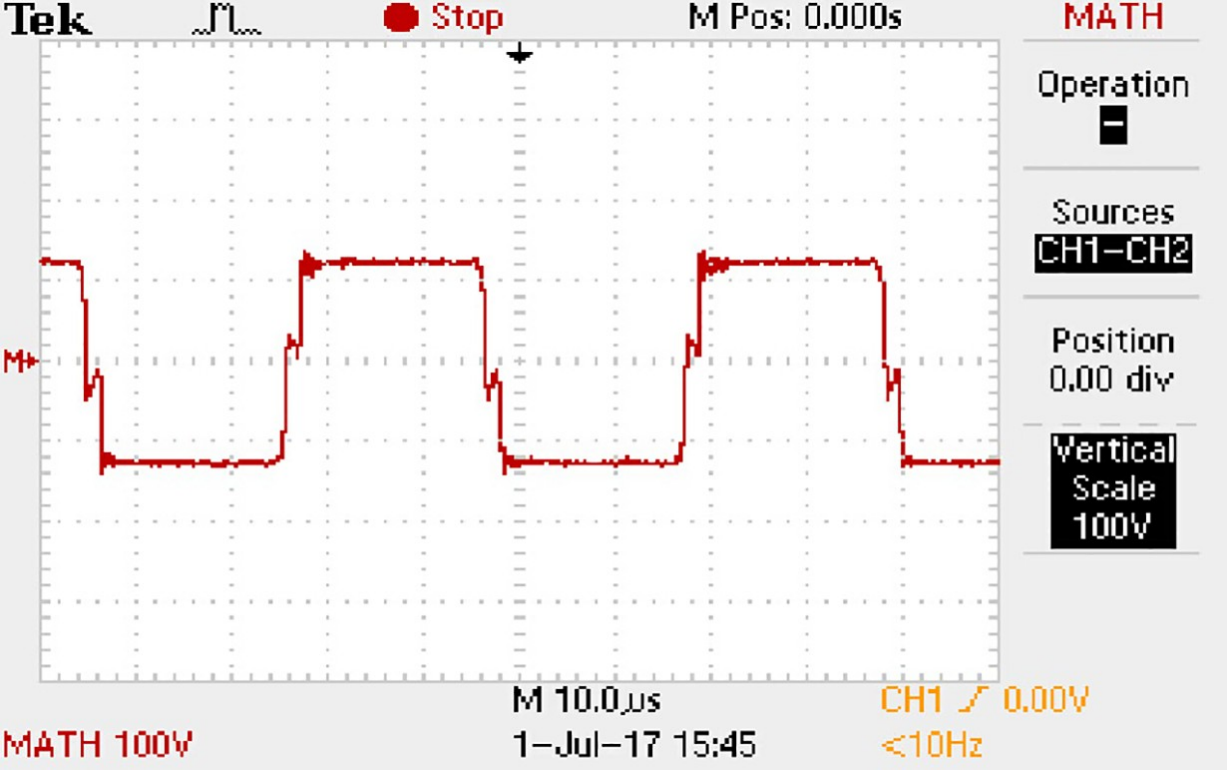
Measured waveforms.

**Fig 60:**
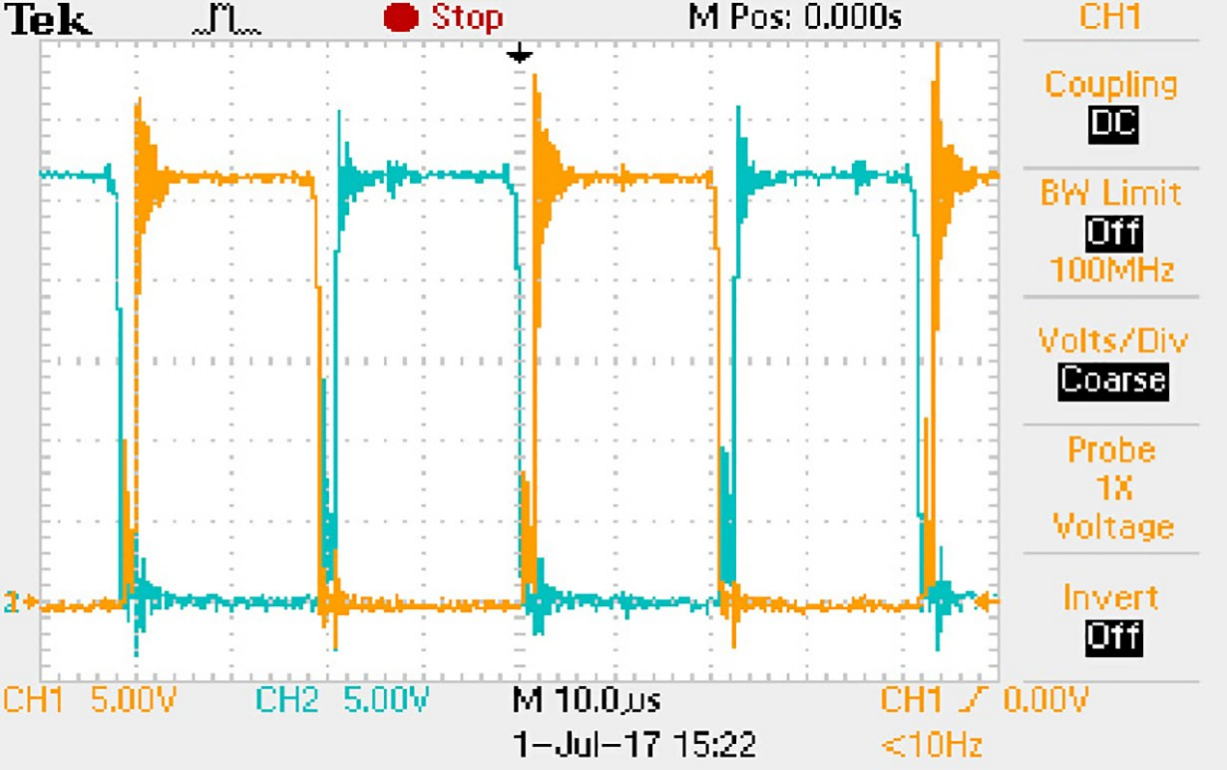
Measured waveforms.

**Fig 61:**
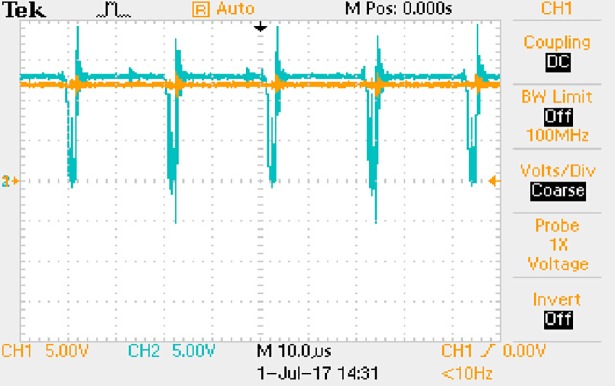
Measured waveforms.

**Fig 62:**
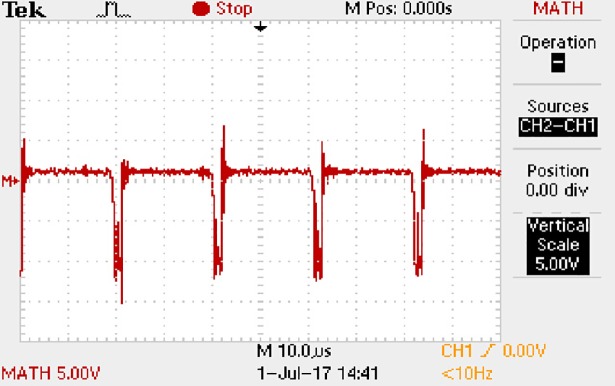
Measured waveforms.

The measured waveforms can be seen from Figs 57–62.

In Fig 57, the CH1 means the drain-source voltage of Q1 and the CH2 means the gate-source voltage of Q1.

In Fig 58, the CH1 means the drain-source voltage of Q4 and the CH2 means the gate-source voltage of Q4.

In Fig 59, the waveform means midpoint voltage of two arms.

In Fig 60, the CH1 means the reverse voltage of D2 and the CH2 means the reverse voltage of D1.

In Fig 61, the CH1 means the output voltage and the CH2 means the total voltage corresponding to the secondary side of transformer and the Schottky rectifier diode.

In Fig 62, the waveform means the voltage of filter inductor.

Based on Figs 57–62, the following results can be obtained.

1) The drain-source voltage of Q1 and Q4 can be almost estimated as zero when they are turning-on.

2) The approximate amplitude of the midpoint voltage of two arms is 120.0V. And the approximate amplitude of the voltage of filter inductor is 1.2V.

3) The output voltage is 12.0V.

4) The rise time and fall time of U_AB_ are both 3μs.

5) The fluctuation degree of the reverse bias voltage related to the D1 and D2 is small.

6) The conduction time of each MOSFET in each switching period t_on_ is 19μs.

## 5 Interpretation of results

### 5.1 Discussion of results

**1.** Observing the gate-source voltage of Q1 and Q4, and the drain-source voltage of Q1 and Q4 in the following indicated figures, we can summarize and list the obtained results. Furthermore, the related conclusion is drawn as follows. The waveforms of Q3 and Q2 are not shown because they are the same as that of Q1 and Q4 respectively.

#### (A) Indicated figures

**Typical model:** Fig 12, Fig 18, and Fig 23.

**Proposed model:** Fig 30, Fig 35, and Fig 40.

**Experiment:** Fig 44, Fig 45, Fig 50, Fig 51, Fig 57, and Fig 58.

#### (B) Results

Whether the drain-source voltage of Q1 and Q4 can be estimated as zero when they are turning-on is regarded as the observed results. The respective situations are summarized and presented in Table 2 and Table 3.

**Table 2:** Situations of Q1.

	0.1A	0.5A	1A
Typical model	Yes	Yes	Yes
Proposed model	Yes	Yes	Yes
Experiment	Yes	Yes	Almost possible

**Table 3:** Situations of Q4.

	0.1A	0.5A	1A
Typical model	Yes	Yes	Yes
Proposed model	Yes	Yes	Yes
Experiment	Yes	Yes	Almost possible

#### (C) Conclusion

Whether it is the model or the actual converter, it all can prove that MOSFETs can realize zero voltage turning-on when PWM strategy is used. Under the light load condition, switching loss of converter is mainly the turning-off loss of MOSFETs.

**2.** Observing the midpoint voltage of two arms and the voltage of filter inductor in the following indicated figures, we can summarize and list the obtained results. Furthermore, the related conclusion is drawn as follows.

#### (A) Indicated figures

**Typical model:** Fig 13, Fig 15, Fig 19, Fig 21, Fig 24, and Fig 26.

**Proposed model:** Fig 31, Fig 33, Fig 36, Fig 38, Fig 41, and Fig 43.

**Experiment:** Fig 46, Fig 49, Fig 52, Fig 56, Fig 59, and Fig 62.

#### (B) Results

The approximate amplitude of the midpoint voltage of two arms and the approximate amplitude of the voltage of filter inductor can be treated as the key characteristic of respective voltage. They are summarized and presented in Table 4 and Table 5.

**Table 4:** Approximate amplitudes of the midpoint voltage of two arms (V).

	0.1A	0.5A	1A
Typical model	115.0	120.0	120.0
Proposed model	115.0	120.0	120.0
Experiment	115.0	120.0	120.0

**Table 5:** Approximate amplitudes of the voltage of filter inductor (V).

	0.1A	0.5A	1A
Typical model	1.0	1.5	1.5
Proposed model	1.0	1.5	1.5
Experiment	0.8	1.2	1.2

#### (C) Conclusion

The differences of approximate amplitude between the two models and the actual converter are all small. It is reasonable that the equivalent resistance R_core_ in the typical model and the proposed model can be estimated by Fig 3 and Fig 4 when the full-bridge works under the light load condition.

**3.** Observing the input current of the converter in the following indicated figures, we can summarize and list the obtained results. Furthermore, the related conclusion is drawn as follows.

#### (A) Indicated figures

**Typical model:** Fig 13, Fig 19, and Fig 24.

**Proposed model:** Fig 31, Fig 36, and Fig 41.

**Experiment:** Fig 17, Fig 28, and Fig 53.

#### (B) Results

Whether the respective input current can be treated as triangular waveform and the rise time is close to the half of switching period *T*/2 is regarded as the observed results. The respective situations are summarized and presented in Table 6.

**Table 6:** Situations of input current.

	0.1A	0.5A	1A
Typical model	Yes	Yes	Almost possible
Proposed model	Yes	Yes	Almost possible
Experiment	Yes	Yes	Almost possible

#### (C) Conclusion

It is reasonable that the input current is approximately expressed by Fig 11 when the full-bridge works under the light load condition. The I_in_ in Fig 11 can be used to calculate the power efficiency of converter.

**4.** Observing the input current and the output voltage in the following indicated figures originated from the typical model, we can summarize and list the obtained results. Furthermore, the related conclusion is drawn as follows.

#### (A) Indicated figures

**Input current:** Fig 13, Fig 19, and Fig 24.

**Output voltage:** Fig 15, Fig 21, and Fig 26.

#### (B) Results

The average input current and the output voltage can be considered as their respective key characteristic. They are summarized and presented in Table 7.

**Table 7:** Average input current and output voltage in the typical model.

	0.1A	0.5A	1A
Average input current	0.0171A	0.0620A	0.10996A
Output voltage	12.016V	12.004V	12.030V

#### (C) Conclusion

When the levels of output current I_o_ are 0.1A, 0.5A, and 1A, the related power efficiency can be deduced from this table. According to the (12), the corresponding values of power efficiency are 61.33%, 80.70%, and 91.40% respectively.

**5.** Observing the input current and the output voltage in the following indicated figures originated from the proposed model, we can summarize and list the obtained results. Furthermore, the related conclusion is drawn as follows.

#### (A) Indicated figures

**Input current:** Fig 31, Fig 36, and Fig 41.

**Output voltage:** Fig 33, Fig 38, and Fig 43.

#### (B) Results

The average input current and the output voltage can be considered as their respective key characteristic. They are summarized and presented in Table 8.

**Table 8:** Average input current and output voltage in the proposed model.

	0.1A	0.5A	1A
Average input current	0.0202A	0.0682A	0.12162A
Output voltage	12.001V	12.089V	12.058V

#### (C) Conclusion

When the levels of output current I_o_ are 0.1A, 0.5A, and 1A, the related power efficiency can be deduced from this table. According to the (12), the corresponding values of power efficiency are 51.67%, 74.41%, and 83.02% respectively.

**6.** Observing the measured input current and the output voltage in the following indicated figures originated from the experiment, we can summarize and list the obtained results. Furthermore, the related conclusion is drawn as follows.

#### (A) Indicated figures

**Input current:** Fig 17, Fig 53, and Fig 28.

**Output voltage:** Fig 48, Fig 55, and Fig 61.

#### (B) Results

The average input current and the output voltage can be considered as their respective key characteristic. They are summarized and presented in Table 9.

**Table 9:** Average input current and output voltage in the experiment.

	0.1A	0.5A	1A
Average input current	0.0210A	0.0704A	0.125A
Output voltage	12.0V	12.0V	12.0V

#### (C) Conclusion

When the levels of output current I_o_ are 0.1A, 0.5A, and 1A, the related power efficiency can be deduced from this table. According to the (12), the corresponding values of power efficiency are 49.69%, 71.02%, and 80.00% respectively.

### 5.2 Comparisons of indexes

Approximation degree between the two theoretical power loss models and the actual converter is investigated according to the indexes designed in the second section.

#### 5.2.1 Local indexes

**1.** Observing the rise time and fall time of U_AB_ in the following indicated figures, we can summarize and list the obtained results in Table 10 and Table 11. Furthermore, the related conclusion is drawn as follows.

**(A) Indicated figures**:

**Typical model:** Fig 13, Fig 19, and Fig 24.

**Proposed model:** Fig 31, Fig 36, and Fig 41.

**Experiment:** Fig 46, Fig 52, and Fig 59.

**(B) Results**:

According to the corresponding measured data, the rise time and fall time of U_AB_ are summarized and presented in the following tables respectively.

**Table 10:** Rise time of U_AB_ (μs).

	0.1A	0.5A	1A
Typical model	4.1927	3.7849	2.7418
Proposed model	3.5358	4.2559	3.0271
Experiment	3	5	3

**Table 11:** Fall time of U_AB_ (μs).

	0.1A	0.5A	1A
Typical model	4.2428	3.9637	2.5332
Proposed model	3.5684	4.0763	2.9455
Experiment	3	5	3

The aforementioned experimental data are also estimated according to the corresponding storage data in the Tektronix oscilloscope TDS2012B.

**(C) Conclusion**:

The differences of the data between the proposed model and the actual converter are all smaller than that between the typical model and the actual converter when the converter works under the light load condition.

**2.** Observing the reverse bias voltage related to the D1 and D2, the total voltage corresponding to the secondary side of transformer and the Scotty rectifier diode in the following indicated figures, we can summarize and list the obtained results. Furthermore, the related conclusion is drawn as follows.

**(A) Indicated figures**:

**Typical model:** Fig 14, Fig 15, Fig 20, Fig 21, Fig 25, and Fig 26.

**Proposed model:** Fig 32, Fig 33, Fig 37, Fig 38, Fig 42, and Fig 43.

**Experiment:** Fig 47, Fig 48, Fig 54, Fig 55, Fig 60, and Fig 61.

**(B) Results**:

The fluctuation degree of the reverse bias voltage can be treated as the key characteristic of respective voltage. For convenience, the “Large” and “Small” are used to judge the fluctuation degree under the same output current. The respective situations are summarized and presented in Table 12.

**Table 12:** Situations of fluctuation degree.

	0.1A	0.5A	1A
Typical model	Large	Large	Large
Proposed model	Small	Small	Small
Experiment	Small	Small	Small

**(C) Conclusion**:

It can be qualitatively concluded that the differences of fluctuation degree between the proposed model and the actual converter are all smaller than that between the typical model and the actual converter when the converter works under the light load.

Through the analyses of aforementioned two local indexes, the following inference can be known. For the proposed model, it is reasonable and effective that the influence of the antiparallel freewheeling diodes at the arms of full-bridge circuit is considered and the barrier junction capacitance of Schottky diode in the rectifier circuit is neglected. Therefore, to some extent, it can be qualitatively judged that the proposed model is closer to the actual converter than the typical model.

#### 5.2.2 Global indexes

On the bases of aforementioned inferences about the two models, the conduction time of each MOSFET in each switching period t_on_ and the power efficiency of converter η are deeply compared. The range for the levels of output current I_o_ are expanded to 0.1A, 0.2A, …, and 1A respectively. The results are summarized and shown in Table 13 and Table 14.

**Table 13:** Time t_on_ (μs).

	Typical model	Proposed model	Experiment
0.1A	8	9	8
0.2A	7	8	8
0.3A	8	9	9
0.4A	10	10	10
0.5A	11	12	12
0.6A	13	13	13
0.7A	14	15	15
0.8A	15	16	17
0.9A	16	17	17
1A	17	19	19

**Table 14:** Power efficiency η.

	Typical model	Proposed model	Experiment
0.1A	61.33%	51.67%	49.69%
0.2A	67.10%	60.83%	56.34%
0.3A	71.62%	64.20%	62.50%
0.4A	77.46%	72.85%	68.85%
0.5A	80.70%	74.41%	71.02%
0.6A	83.33%	75.89%	73.35%
0.7A	85.57%	78.59%	75.35%
0.8A	86.55%	77.97%	76.19%
0.9A	88.06%	79.35%	77.59%
1A	91.40%	83.02%	80.00%

For the time t_on_ in Table 13 and the power efficiency η in Table 14, it is the fact that the differences between the proposed model and the actual converter are almost smaller than that between the typical model and the actual converter. Therefore, for the proposed model shown in Fig 6, the equivalent turning-off loss resistance R_switch_, the equivalent core loss resistance of inductive components R_core_, and the other equivalent conduction loss resistances of converter are reasonable and effective. Even it can be quantitatively judged that the proposed model can better formulate the actual converter than the typical model.

In terms of the local indexes and the global indexes, we can obtain the following summary according to the comprehensive comparisons between the two models and the actual converter. The conclusion is that the proposed model shown in Fig 6 is more approximate to the actual converter than the typical model under the light load condition.

## 6 Conclusion

In this paper, the full-bridge converter works under the light load condition that the filter inductor current operates in the CCM. It is taken as the research background. Power loss models of converter are established on the bases of investigated condition. Firstly, the typical model has been developed according to the existing achievements. The PWM strategy is applied in the actual converter. Secondly, the antiparallel freewheeling diodes are added at the arms of full-bridge and each barrier junction capacitance of Schottky diode in the rectifier circuit is neglected. Meanwhile, the theoretical results of typical model and actual input current under the minimum and maximum output current are fully utilized. Then, the proposed model is founded by compensating and correcting the equivalent turning-off loss resistance, the equivalent core loss resistance of inductive components, and the equivalent conduction loss resistance of converter. The lastly but important, the two power loss model are compared with the actual converter respectively in accordance with the local indexes and the global indexes. Based on the comparisons of results, it can be concluded that the proposed model is closer to the actual converter than the typical model under the light load condition. This lays a foundation on the research for the power efficiency of full-bridge converter under light load.

## 7 Appendix

**A Isolated Transformer**

1) Magnetic core: EE65

2) Effective cross sectional areas: 535mm^2^

3) Effective volumes: 78700mm^3^

4) Number of turns at primary side: 80

5) Number of turns at each secondary side: 9.5

6) Resistance coefficient of winding: 0.01749Ω.m/mm^2^

7) Length of winding at primary side: 8.4m

8) Radius of enameled wire at primary side: 0.25mm

9) Number of strands referred to the enameled wire at primary side: 3

10) Length of winding at each secondary side: 1m

11) Radius of enameled wire at each secondary side: 0.25mm

12) Number of strands referred to the enameled wire at each secondary side: 7

**B Filter Inductor**

1) Magnetic core: EE55

2) Effective cross sectional areas: 354mm^2^

3) Effective volumes: 43700mm^3^

4) Number of turns: 30

5) Resistance coefficient of winding: 0.01749Ω.m/mm^2^

6) Length of winding: 2.62m

7) Radius of enameled wire: 0.25mm

8) Number of strands referred to enameled wire: 7

## Supporting information

S1 File Parameters of actual converter. (PDF)

## References

[1] Hwang DH, and Lee WC. Global maximum power point tracking method of photovoltaic array using boost converter. *Trans*. *KIEE*. 2018; 67(2): 216–223. 10.5370/KIEE.2018.67.2.216

[2] Huang C, Wang L, Yeung RSC, Zhang ZJ, Chung HSH, and Bensoussan A. A prediction model-guided Jaya algorithm for the PV system maximum power point tracking. *IEEE Trans*. *Sustain*. *Energy*. 2018; 9(1): 45–55. 10.1109/TSTE.2017.2714705

[3] Batzelis EI, Kampitsis GE, and Papathanassiou SA. Power reserves control for PV systems with real-time MPP estimation via curve fitting. *IEEE Trans*. *Sustain*. *Energy*. 2017; 8(3): 1269–1280. 10.1109/TSTE.2017.2674693

[4] Sampaio LP, da Silva SAO. Graphic computational platform integrated with an electronic emulator dedicated to photovoltaic systems teaching. *IET Power Electron*. 2017; 10(14): 1982–1992. 10.1049/iet-pel.2016.1018

[5] Mira MC, Zhang Z, Knott A, and Andersen MAE. Analysis, design, modeling, and control of an interleaved-boost full-bridge three-port converter for hybrid renewable energy systems. *IEEE Trans*. *Power Electron*. 2017; 32(2): 1138–1155. 10.1109/TPEL.2016.2549015

[6] Roche M, Shabbir W, and Evangelou SA. Voltage control for enhanced power electronic efficiency in series hybrid electric vehicles. *IEEE Trans*. *Veh*. *Technol*. 2017; 66(5): 3645–3658. 10.1109/TVT.2016.2599153

[7] Lai CM, Cheng YH, Hsieh MH, and Lin YC. Development of a bidirectional DC/DC converter with dual-battery energy storage for hybrid electric vehicle system. *IEEE Trans*. *Veh*. *Technol*. 2018; 67(2): 1036–1052. 10.1109/TVT.2017.2763157

[8] Buticchi G, Costa LF, Barater D, Liserre M, and Amarillo ED. A quadruple active bridge converter for the storage integration on the more electric aircraft. *IEEE Trans*. *Power Electron*. 2018; 33(9): 8174–8186. 10.1109/TPEL.2017.2781258

[9] Lin N, and Dinavahi V. Dynamic electro-magnetic-thermal modeling of MMC-based DC-DC converter for real-time simulation of MTDC grid. *IEEE Trans*. *Power Del*. 2018; 33(3): 1337–1347. 10.1109/TPWRD.2017.2774806

[10] Tiwari SK, Singh B, and Goel PK. Design and control of microgrid fed by renewable energy generating sources. *IEEE Trans*. *Ind*. *Appl*. 2018; 54(3): 2041–2050. 10.1109/TIA.2018.2793213

[11] Prasanna UR, and Rathore AK. Extended range ZVS active-clamped current-fed full-bridge isolated DC/DC converter for fuel cell applications: analysis, design, and experimental results. *IEEE Trans*. *Ind*. *Electron*. 2013; 60(7): 2661–2672. 10.1109/TIE.2012.2194977

[12] Lai YS, and Su ZJ. Novel on-line maximum duty point tracking technique to improve two-stage server power efficiency and investigation into its impact on hold-up time. *IEEE Trans*. *Ind*. *Electron*. 2014; 61(5): 2252–2263. 10.1109/TIE.2013.2273479

[13] Pahlevaninezhad M, Drobnik J, Jain PK, and Bakhshai A. A load adaptive control approach for a zero-voltage-switching DC/DC converter used for electric vehicles. *IEEE Trans*. *Ind*. *Electron*. 2012; 59(2): 920–933. 10.1109/TIE.2011.2161063

[14] Jang Y, and Jovanovic MM. Light-load efficiency optimization method. *IEEE Trans*. *Power Electron*. 2010; 25(1): 67–74. 10.1109/TPEL.2009.2024419

[15] Liang M, Zheng TQ, and Li Y. An improved analytical model for predicting the switching performance of SiC MOSFETs. *J*. *Power Electron*. 2016; 16(1): 374–387. 10.6113/JPE.2016.16.1.374

[16] Zhang TZ, Fu JY, Qian QS, Sun WF, and Lu SL. Dead-time for zero-voltage-switching in battery chargers with the phase-shifted full-bridge topology: comprehensive theoretical analysis and experimental verification. *J*. *Power Electron*. 2016; 16(2): 425–435. 10.6113/JPE.2016.16.2.425

[17] Lai YS, Su ZJ, and Chang YT. Novel phase-shift control technique for full-bridge converter to reduce thermal imbalance under light-load condition. *IEEE Trans*. *Ind*. *Appl*. 2015; 51(2): 1651–1659. 10.1109/TIA.2014.2347454

[18] Wang HY, Shang M, and Khaligh A. A PSFB-based integrated PEV onboard charger with extended ZVS range and zero duty cycle loss. *IEEE Trans*. *Ind*. *Appl*. 2017; 53(1): 585–595. 10.1109/TIA.2016.2615034

[19] Hasari SAS, Salemnia A, and Hamzeh M. Applicable method for average switching loss calculation in power electronic converters. *J*. *Power Electron*. 2017; 17(4): 1097–1108. 10.6113/JPE.2017.17.4.1097

[20] Kim DY, Kim CE, and Moon GW. Variable delay time method in the phase-shifted full-bridge converter for reduced power consumption under light load conditions. *IEEE Trans*. *Power Electron*. 2013; 28(11): 5120–5127. 10.1109/TPEL.2013.2237926

[21] Kim JW, Kim DY, Kim CE, and Moon GW. A simple switching control technique for improving light load efficiency in a phase-shifted full-bridge converter with a server power system. *IEEE Trans*. *Power Electron*. 2014; 29(4): 1562–1566. 10.1109/TPEL.2013.2279549

[22] Tian JS, Gao JX, and Zhang YM. Design of a novel integrated L-C-T for PSFB ZVS converters. *J*. *Power Electron*. 2017; 17(4): 905–913. 10.6113/JPE.2017.17.4.905

[23] Cho KM, Kim YD, Cho IH, and Moon GW. Transformer integrated with additional resonant inductor for phase-shift full-bridge converter with primary clamping diodes. *IEEE Trans*. *Power Electron*. 2012; 27(5): 2405–2414. 10.1109/TPEL.2011.2106514

[24] Hatakeyama T, and Onda K. Core loss estimation of various materials magnetized with the symmetrical/asymmetrical rectangular voltage. *IEEE Trans*. *Power Electron*. 2014; 29(12): 6628–6635. 10.1109/TPEL.2014.2306755

[25] Kim JH, Kim CE, Kim JK, Lee JB, and Moon GW. Analysis on load-adaptive phase-shift control for high efficiency full-bridge *LLC* resonant converter under light-load conditions. *IEEE Trans*. *Power Electron*. 2016; 31(7): 4942–4955. 10.1109/TPEL.2015.2462077

[26] Fei C, Ahmed MH, Lee FC, and Li Q. Two-stage 48V-12V/6V-1.8V voltage regulator module with dynamic bus voltage control for light-load efficiency improvement. *IEEE Trans*. *Power Electron*. 2017; 32(7): 5628–5636. 10.1109/TPEL.2016.2605579

[27] Yadav GNB, and Narasamma NL. An active soft switched phase-shifted full-bridge DC–DC converter: analysis, modeling, design, and implementation. *IEEE Trans*. *Power Electron*. 2014; 29(9): 4538–4550. 10.1109/TPEL.2013.2284780

[28] Lai YS, Su ZJ, and Chen WS. New hybrid control technique to improve light load efficiency while meeting the hold-up time requirement for two-stage server power. *IEEE Trans*. *Power Electron*. 2014; 29(9): 4763–4775. 10.1109/TPEL.2013.2283747

[29] Hirose T, Takasaki M, and Ishizuka Y. A power efficiency improvement technique for a bidirectional dual active bridge DC-DC converter at light load. *IEEE Trans*. *Ind*. *Appl*. 2014; 50(6): 4047–4055. 10.1109/TIA.2014.2327147

[30] Zeng HL, Gonzalez-Santini NS, Yu YD, Yang ST, and Peng FZ. Harmonic burst control strategy for full-bridge series-resonant converter-based EV charging. *IEEE Trans*. *Power Electron*. 2017; 32(5): 4064–4073. 10.1109/TPEL.2016.2589942

[31] Lai YS, and Su ZJ. New integrated control technique for two-stage server power to improve efficiency under the light-load condition. *IEEE Trans*. *Ind*. *Electron*. 2015; 62(11): 6944–6954. 10.1109/TIE.2015.2436872

