# Supplementary material for: An improved power loss model of full-bridge converter under light load condition
Source: PLoS One. 2018 Dec 6;13(12):e0208239. doi: 10.1371/journal.pone.0208239 (PMC6283570; doi:10.1371/journal.pone.0208239)
Supplement: S1 File — (PDF) [file pone.0208239.s001.pdf]

Parameters of actual converter

Input voltage U_{in}	Case 1: 115V; Case 2: 120V
Output voltage U_o	12V
Current under full load	10A
MOSFET	IRFP460
Antiparallel freewheeling diode	MUR3060WT
Leakage inductance	600μH
Excitation inductance	11.5mH
Turns ratio	8.42:1:1
Rectifier diode	V50100PW
Filter inductor	250μH
Filter capacitor	470μF
Switching frequency	24kHz
Driven resistors $R_{11}$to R_{14}	6.2Ω
Driven resistors R_{21} to R_{24}	10kΩ
Zener diodes V_{z1} to V_{z4}	IN4744A

A Isolated Transformer

1) Magnetic core: EE65

2) Effective cross sectional areas: 535mm^2

3) Effective volumes: 78700mm^3

4) Number of turns at primary side: 80

5) Number of turns at each secondary side: 9.5

6) Resistance coefficient of winding: 0.01749Ω.m/mm^2

7) Length of winding at primary side: 8.4m

8) Radius of enameled wire at primary side: 0.25mm

9) Number of strands referred to the enameled wire at primary side: 3

10) Length of winding at each secondary side: 1m

11) Radius of enameled wire at each secondary side: 0.25mm

12) Number of strands referred to the enameled wire at each secondary side: 7

B Filter Inductor

1) Magnetic core: EE55

2) Effective cross sectional areas: 354mm^2

3) Effective volumes: 43700mm^3

4) Number of turns: 30

5) Resistance coefficient of winding: 0.01749Ω.m/mm^2

6) Length of winding: 2.62m

7) Radius of enameled wire: 0.25mm

8) Number of strands referred to enameled wire: 7
